# Simultaneous Spin Coating and Ring-Opening Metathesis Polymerization for the Rapid Synthesis of Polymer Films

**DOI:** 10.1021/acsami.4c00211

**Published:** 2024-03-22

**Authors:** Zane J. Parkerson, Liudmyla Prozorovska, Matthew P. Vasuta, Tyler D. Oddo, G. Kane Jennings

**Affiliations:** † Department of Chemical and Biomolecular Engineering, Vanderbilt University, Nashville, Tennessee 37235, United States; ‡ Interdisciplinary Materials Science Program, Vanderbilt University, Nashville, Tennessee 37235, United States

**Keywords:** ROMP, thin films, copolymers, membranes, pervaporation

## Abstract

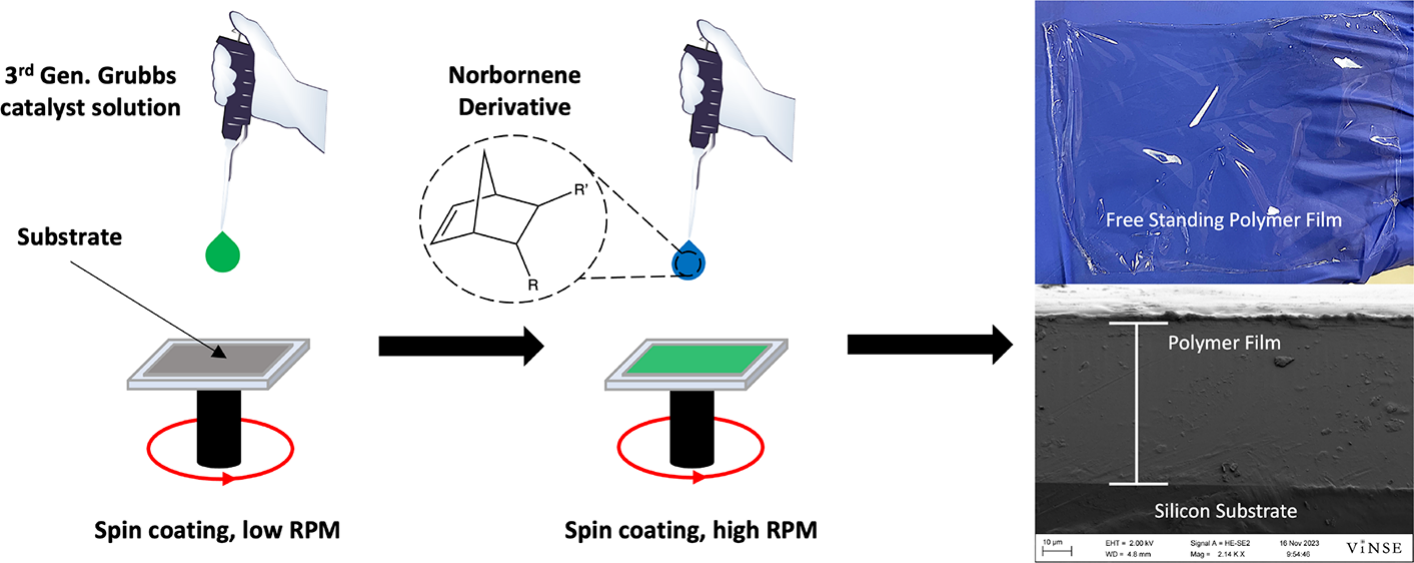

We report a highly controlled technique for the synthesis of polymer films atop a substrate by combining spin coating with ring-opening metathesis polymerization (ROMP), herein termed spin coating ROMP (scROMP). The scROMP approach combines polymer synthesis and deposition into one process, fabricating films of up to 36 cm^2^ in under 3 min with orders-of-magnitude reduction in solvent usage. This method can convert numerous norbornene-type molecules into homopolymers and random copolymers as uniform films on both porous and nonporous substrates. Film thickness can be varied from a few hundred nanometers to a few tens of micrometers based on spin speed and monomer concentration. The resulting polymers possess high *M*_W_ (>100 kDa) and low polydispersity (PDI) (<1.2) values that are similar to ROMP polymers made in solution. We also devise a model to investigate the balance between convective monomer spin-off and polymer growth from the surface, which allows the determination of critical kinetic parameters for scROMP. Finally, translation of scROMP to porous supports enables the synthesis of thin film composite membranes that demonstrate the ability to dehydrate ethanol by pervaporation.

## Introduction

Polymer films have broad applications in surface modification, such as to mitigate corrosion,^1^ alter wetting,^2,3^ friction,^4^ and/or adhesion^5,6^ and separate chemical species.^7−9^ For these applications, the polymers are often synthesized in solution and then deposited by casting, spin coating, or grafting the chains onto the substrate.^10^ As bulk polymer synthesis and the associated separations can be slow, the process of evaluating many different polymer film compositions for a particular application is often arduous and lengthy. Surface-initiated polymerization, or the grafting-from approach, can be a much faster route to ultrathin polymer films with simple rinsing steps as separations.^11^ However, the covalent attachment of the initiator or catalyst to the substrate reduces the kinetics of polymerization versus that in the bulk^12,13^ and hinders access to thicker films that are often needed in these applications.

**Scheme 1:**
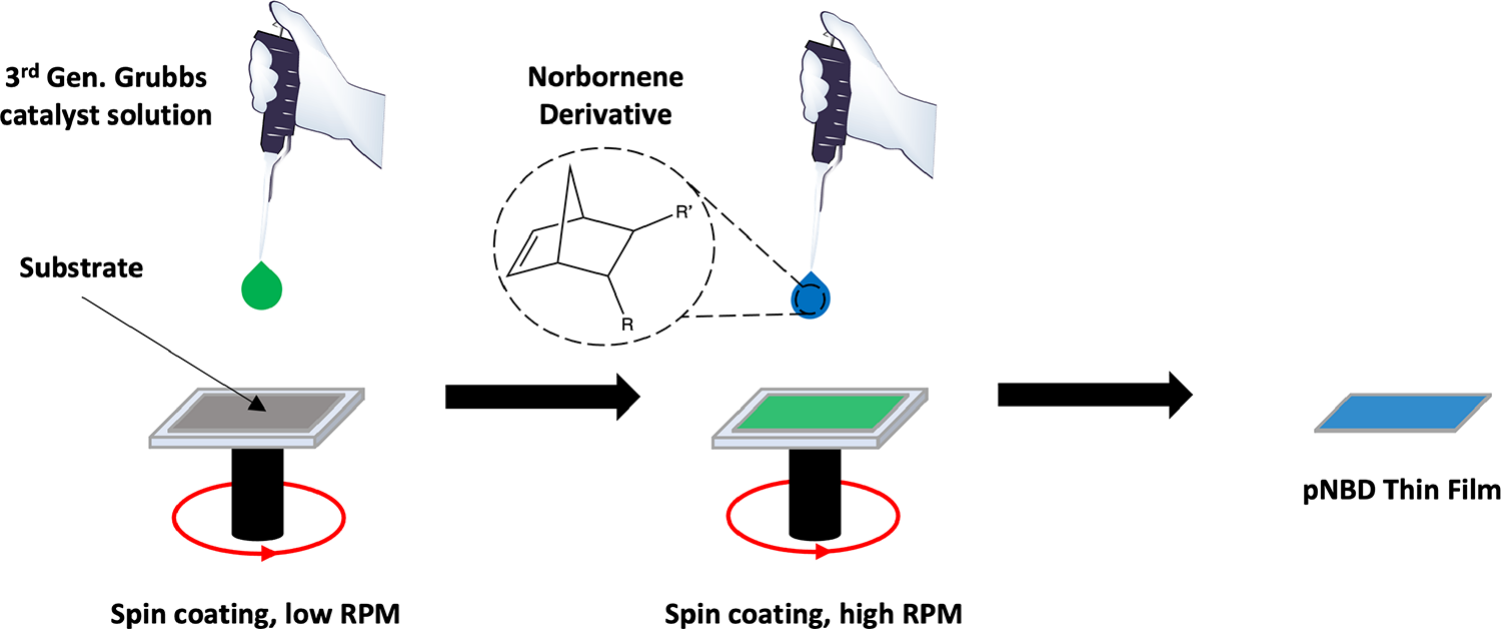
Schematic of the scROMP Process in which a Polymer Film is Synthesized atop a Substrate by Spinning on a Catalyst Followed by a Monomer

We hereby merge polymer synthesis and film deposition into a single and rapid process. Specifically, we combine spin coating with ring-opening metathesis polymerization (ROMP) into a process that we term scROMP (Scheme 1).

The process consists of spinning on a small volume of Grubbs third generation catalyst (G3) in dichloromethane (DCM) onto a substrate and then spinning on a monomer, either as a neat liquid or in a solvent. This process results in polymer films in under 2 min of spinning time, using less than 1 mL of solvent and enabling many different polymer film compositions to be synthesized in a short period of time. The scROMP technique possesses advantages over spin coating alone. Spin coating applications frequently apply a polymer solution to a substrate, whereas scROMP synthesizes the polymer film from the substrate and thereby removes the prior solvent-intensive steps of bulk polymer synthesis and dissolution required for spin coating. Further, scROMP enables deposition of films that are not sufficiently soluble to enable spin coating or casting.

The use of ROMP here is strategic due to its rapid initiation,^14,15^ especially with G3 as the catalyst, its ability to produce polymers with low polydispersity index (PDI) through a “living” process, and its relatively high tolerance of oxygen and other components in the ambient air.^16,17^ ROMP is a chain-growth polymerization method that converts cyclic olefin monomers to monodisperse polymers with unsaturated backbones. ROMP is driven by the relief of ring strain, which overcomes entropic penalties associated with polymerization. As such, monomers with considerable ring strain, such as norbornenes, react efficiently and to completion.^18^ This technique grew popular with Robert Grubbs’ development of Ru-based catalysts^19^ that are selective toward olefins over other functional groups and tolerant of ambient conditions. Numerous catalysts enable living ROMP; therefore, ROMP has emerged as a versatile living polymerization technique that can tolerate other functional groups.

The tolerance of ambient conditions when using Ru-based catalysts has been illustrated by carrying out polymerizations in aqueous media^16,17^ and in ambient laboratory conditions^20,21^ using Ru-based catalysts such as G3. ROMP polymers have been synthesized through novel methods with great success. Walsh et al.^22^ utilized G3 to synthesize poly(norbornene) (pNB) at specific *M*_W_ with low PDI through a flow-controlled polymerization approach. Specifically, the authors adjusted the flow rate of monomer and G3 through a flow reactor to synthesize polymers possessing *M*_W_ = 4–1000 kDa and PDI = 1.02–1.11. Alzate-Sanchez et al.^23^ devised a unique way to carry out ROMP in a solventless method. Their method, termed frontal ROMP (FROMP), uses the exothermic nature of ROMP to thermally activate the initiator, Grubbs second generation catalyst (G2), and produce a moving polymerization front. In their process, the monomer and G2 are combined and spread onto a glass slide. Contacting a soldering iron to the reaction mixture generates a moving polymerization reaction front that converts the liquid monomer into the solid polymer over short time spans. The FROMP process was used to generate a polymer with *M*_W_ = 116 kDa and PDI = 1.31 in just 45 s, demonstrating that ROMP can be carried out in a solventless process and produce well-defined polymers rapidly. Lastly, Escobar et al. demonstrated that the combination of bound and mobile G2 catalysts can polymerize a contacting monomer phase to produce films exceeding 1 μm in thickness with a micropatterned topography.^24,25^ In this process, molds with varying surface patterns were coated with a G2 catalyst and filled with a neat liquid monomer before being interfaced to a smooth substrate that contained an immobilized layer of G2. Peeling away the mold resulted in a film that possesses the microstructures present on the mold, such as pyramids^24^ or imprinted superhydrophobic leaves.^25^

In this work, we introduce the scROMP technique and demonstrate its capabilities to synthesize a range of polymer films using various cyclic olefins and show that spin speed can be used to control the thicknesses of the resulting films. We report a simplified model of the scROMP process and use this model to quantify the effects of spin speed on the kinetic rate constant for film propagation. We also discuss the effects of catalyst loading and spin speed on the average chain molecular weight and polydispersity. Additionally, we demonstrate the capability of the scROMP approach to generate random copolymer films that possess some of the favorable properties of each monomer’s homopolymer. Finally, we showcase the potential for thin film composite (TFC) membranes to be synthesized by conducting the scROMP technique onto a porous polymer support to generate a dense outer “skin” that shows selectivity for the dehydration of ethanol.

## Experimental Section

### Materials

Gold shot (99.99%) (J&J Materials) and silicon (100) wafers (University Wafers) were used in the preparation of Au-coated Si/SiO_2_ wafers. *trans*-3,6-Endomethylene-1,2,3,6-tetrahydrophtaloyl chloride (NBDAC) was purchased from Sigma-Aldrich and Santa Cruz Biotechnology. 5-Perfluoro-*n*-alkyl-norbornenes (NBFn; *n* = 4, 6, and 8 perfluorinated carbons) with a 3:1 endo:exo isomer ratio were synthesized as previously reported.^26,27^ Norbornene (NB) and dicyclopentadiene (DCPD) were purchased from Sigma-Aldrich and used as received. DCM, THF, *n*-pentane, acetone, ethyl vinyl ether, and ethanol were purchased from ThermoFisher and used as received. Porous supports of poly(acrylonitrile) (PAN) and poly(ether sulfone) (PES) were purchased from Sterlitech, Inc. with molecular weight cutoffs (MWCO) of 30 and 100 (PAN) and 50 kDa (PES). Prior to use, PAN coupons were cut to a size of 4 or 36 cm^2^ and stored in D.I. water for ∼24 h to remove glycerol, an additive to prevent pore collapse during shipment. After 24 h, coupons were rinsed with D.I. water and stored in fresh D.I. water at room temperature until used. Before use, the films were dried in a stream of nitrogen to remove absorbed water.

### Synthesis of Grubbs Third-Generation Catalyst

Grubbs second-generation catalyst [(H_2_IMes)(PCy_3_)(Cl)_2_Ru = CHPh] (G2) and 3-bromopyridine were used as received from Sigma-Aldrich to synthesize the Grubbs third-generation catalyst [(H_2_IMes)(3-Brpy)_2_(Cl)_2_Ru = CHPh] (G3) as previously described.^14,28^ Briefly, G2 (0.5 g, 0.59 mmol) and 3-bromopyridine (0.94 g, 5.9 mmol) were added to a 20 mL screw cap vial. The mixture was stirred at room temperature for 5 min with a color change from red to bright green observed. After 5 min of stirring, 20 mL of pentane was added to the vial, and a green solid was precipitated. The vial was sealed in air and placed in a freezer overnight. The precipitate was vacuum filtered, washed with 10 mL of pentane four times, and dried under a vacuum to yield G3 as a green powder.

### Preparation of Au-Coated Si/SiO_2_ Wafers

Silicon (100) wafers were rinsed with deionized water and ethanol and dried in a nitrogen stream. Chromium (100 Å) and gold (1250 Å) were evaporated onto the clean silicon wafers at a rate of ≤2 Å/s in a diffusion-pumped chamber at a base pressure of 4 × 10^–6^ Torr. The resulting Au-coated wafers were generally cut into 2 cm × 2 cm samples following evaporation. Prior to use, wafers were rinsed with ethanol, water, and ethanol, and dried in a stream of nitrogen.

### Preparation of Norbornenyl Terminated Surfaces on Silicon and Gold-Coated Wafers

Silicon wafers were cut into 2 cm × 2 cm samples, rinsed with water and ethanol, and dried in a stream of nitrogen. The silicon substrates were then placed in piranha solution (14 mL H_2_SO_4_/6 mL H_2_O_2_) for 30 min to hydroxylate the silicon oxide surface. The substrates were carefully removed from the piranha solution, rinsed with copious amounts of water and ethanol, and dried in a stream of nitrogen. The substrates were then exposed to a 5 mM solution of NBSiCl_3_ in toluene for 1 h to yield a surface-tethered norbornenyl-terminated SAM. Substrates were rinsed with toluene, water, and ethanol, and dried in a stream of nitrogen.

Gold-coated wafers were cut into 2 cm × 2 cm samples after gold evaporation and placed into a 1 mM ethanolic solution of 4-mercapto-1-butanol for 1 h to yield a hydroxyl-terminated self-assembled monolayer (SAM) on the gold surface. The SAM was rinsed with water and then ethanol and dried in a stream of nitrogen. After being dried, the substrate was exposed to a 5 mM solution of NBDAC in DCM for 30 min to generate the acylation product of a surface-tethered norbornenyl group. The norbornenyl-terminated films were rinsed with DCM, water, and ethanol, and then dried in a stream of nitrogen.

### Preparation of Polymer Films

Polymer films were prepared by the scROMP method, as shown in Scheme 1. A solution of G3 in DCM (5 mM) was dispensed on a substrate while the substrate was spinning. For 4 cm^2^ substrates, 200 μL of G3 solution was dispensed on the substrate at 1000 rpm, and for 36 cm^2^ substrates, 400 μL of G3 solution was dispensed on the substrate at 2000 rpm. The substrate was allowed to spin for 30 s to allow for evaporation-facilitated deposition of G3 onto the substrate surface. Immediately following G3 deposition, a volume of monomer, either the neat liquid or in solution, was deposited on the surface at a spin speed ranging from 1000–6000 rpm. Polymerization began as the monomer was dispensed on the surface, and the substrate remained spinning for 60 s. The spin coater was then stopped, and the formed polymer film atop the substrate was rinsed with ethanol, dried in a stream of nitrogen, and characterized.

### Preparation of Copolymer Films

Copolymer films were prepared by using the scROMP method. As with the preparation of homopolymer films, a solution of G3 in DCM was dispensed onto a solid substrate while spinning at 1000 rpm. Following deposition of G3, an amount of a comonomer solution in DCM (total solution concentration of 4 M in pentane for NB-co-NBDAC or 4.5 M in pentane for NB-co-NBF4) was dispensed onto the catalyst-laden surface at a spin speed of 2500 rpm. After 60 s elapsed, the spinning substrate was stopped, and the resulting copolymer film-coated substrate was rinsed with ethanol, dried in a stream of nitrogen, and characterized.

### Characterization

A Rame–Hart goniometer was used to measure sessile, advancing, and receding contact angles at room temperature. A syringe supplied by Rame–Hart was used to dispense, advance, or recede the liquid droplet prior to angle measurement. Each reported value is the average and standard deviation of measurements on at least three different polymer films at three different surface locations.

Attenuated total reflectance Fourier transform infrared spectroscopy (ATR-FTIR) was conducted using a ThermoFisher Nicolet 6700 FT-IR spectrometer equipped with a liquid N_2_-cooled mercury–cadmium–telluride (MCT) detector and Smart iTR ATR attachment with a diamond-crystal plate. Spectra of samples were collected from 4000–600 cm^–1^ through 256 scans at a 2 cm^–1^ resolution.

Polymer film thickness and roughness on Au-coated Si/SiO_2_ were quantified by using a Veeco Dektak 150 profilometer with a diamond tip stylus with a radius of 12.5 μm. The applied force was set to 2 mg, and data were collected at a rate of 0.33 μm/sample. Thickness was quantified by scratching the polymer film such that the underlying substrate was exposed, with care being taken to ensure that the substrate was not marred, and the stylus was run across the scratch. For polymer films made on PAN, an area of the film was masked prior to scROMP using tape. After scROMP was conducted, the tape was removed, revealing a section of the substrate with no polymer film present. Reported film thicknesses represent the average and standard deviation of at least three independent polymer films.

A Zeiss Merlin scanning electron microscope (SEM) containing an Everhart–Thornley secondary electron detector was used to image the top surfaces and cross sections of the polymer films. Prior to imaging, samples were gold-sputtered for 20 s in an argon environment. For the top surface images, samples were mounted to an SEM stub using double-sided carbon tape. For cross-sectional images, samples were mounted to a 90° angle stub using double-sided carbon tape.

Polymer mass average molecular weight (*M*_W_) and polydispersity (PDI) were collected by using a Waters HPLC equipped with a refractive index detector. A Waters Styragel HT 4 column containing 10 μm beads with a 1000 Å pore size and a Waters SunFire Silica Prep Column containing 5 μm beads with a 100 Å pore size were used to characterize polymer *M*_W_. The mobile phase was THF, and polymer films were dissolved in degassed HPLC-grade THF prior to injection. A set of polystyrene standards with *M*_W_ ranging from 370 Da to 500 kDa were used to generate a column calibration curve prior to each sampling. The polystyrene standards were prepared on the same day to prevent aggregation.

Nuclear magnetic resonance (NMR) experiments were acquired on both a 9.3 T Bruker magnet equipped with a Bruker AV console operating at 400.13 MHz and a 14.0 T Bruker magnet equipped with a Bruker AV-III console operating at 600.13 MHz. For Figure S.8, the spectra for films prepared from NB, 3:1 (NB:NBF4), and 1:1 were acquired on the 400 MHz spectrometer, while the spectra for 1:3 and NBF4 were acquired on the 600 MHz spectrometer. The 400 MHz spectrometer was also equipped with a 5 mm pulse field gradient BBFO NMR probe. For 1D ^1^H NMR, experimental conditions included 32,000 data points, 13 ppm sweep width, a recycle delay of 1.5 s, and 16 scans. For the data collected on the 600 MHz spectrometer for 1D ^1^H NMR, experimental conditions included 32,000 data points, 13 ppm sweep width, a recycle delay of 1.5 s, and 64 scans. Multiplicity-edited heteronuclear single quantum coherence (HSQC) experiments were acquired using a 1024 × 256 data matrix and a J(C–H) value of 145 Hz, which resulted in a multiplicity selection delay of 34 ms, a recycle delay of 1.5 s, and 4 scans per increment along with GARP decoupling on ^13^C during the acquisition time (150 ms). The data were processed using a π/2 shifted squared sine window function and displayed with CH/CH_3_ signals phased positive and CH_2_ signals phased negative.

Pervaporation studies were conducted using an Innovator tangential flow cell constructed of PTFE as sold by Sterlitech, Inc. The tested cell has an active surface area of 16 cm^2^. Feed solutions were heated to the desired temperature and pumped to the membrane’s active area via a rotary pump. Membrane permeate was collected by pulling a vacuum using an Edwards 5 vacuum pump and condensing the permeate in a liquid-N_2_-cooled cold trap. Permeate ethanol concentration was quantified using an Atago PAL-34S pocket refractometer calibrated specifically for ethanol/water mixtures.

## Results and Discussion

### Synthesis of Polymer Films

**Figure 1:**
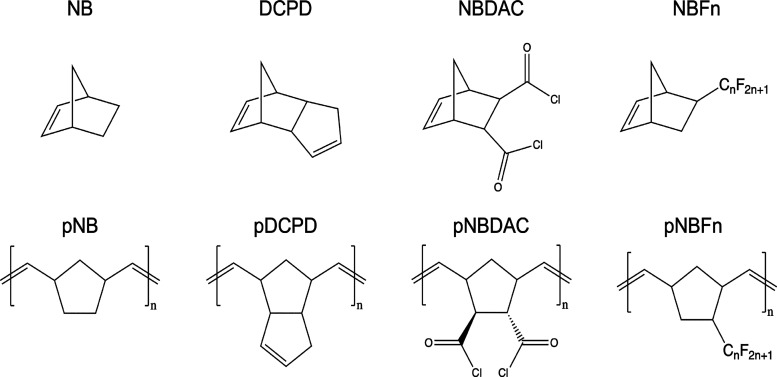
Monomers (top) and polymer repeat unit structures (bottom) of NB, DCPD, NBDAC, and NBFn.

The scROMP approach, shown in Scheme 1, is composed of substrate preparation, spin coating of the G3 solution, and spin coating of the cyclic olefin monomer. The combination of these steps produces a smooth, solid polymer film on the substrate surface. Polymer films synthesized directly on a dense solid substrate, such as a Au-coated Si/SiO_2_ wafer, can be peeled off the substrate’s surface (Figure S.1, right), allowing certain polymer characterization techniques to be performed without influence from the substrate. For coating applications, the surface is premodified to provide a more robust anchoring of the polymer to the substrate. For example, exposure of Si/SiO_2_ to a norbornene-terminated trichlorosilane (NBSiCl_3_)^29^ prior to scROMP provides sites for G3 to attach/bind once the G3 solution is dispensed. The bound catalyst combines with unbound G3 to initiate and propagate a robustly bound polymer film. A similar modification can be performed on Au-coated Si/SiO_2_ wafers using an alkyl thiol monolayer possessing a ROMP-active norbornene terminal group as reported by Faulkner et al. (Figure S.1, left).^26^ On PAN supports with either 30 or 100 kDa *M*_W_ cutoff, the films generated by scROMP are highly stable without the preaddition of surface modifiers (Figure S.2). Similar stability was observed on PES supports. We attribute film stability to limited growth of the polymer in the pores present on the PAN and PES surfaces. To ensure that the scROMP process is suitable for a variety of cyclic olefin monomers, liquid monomers (NBDAC and NBFn) and monomers in solution (NB and DCPD) were used to fabricate the polymer films. The structures of these monomers and their respective repeat units are shown in Figure 1.

**Figure 2:**
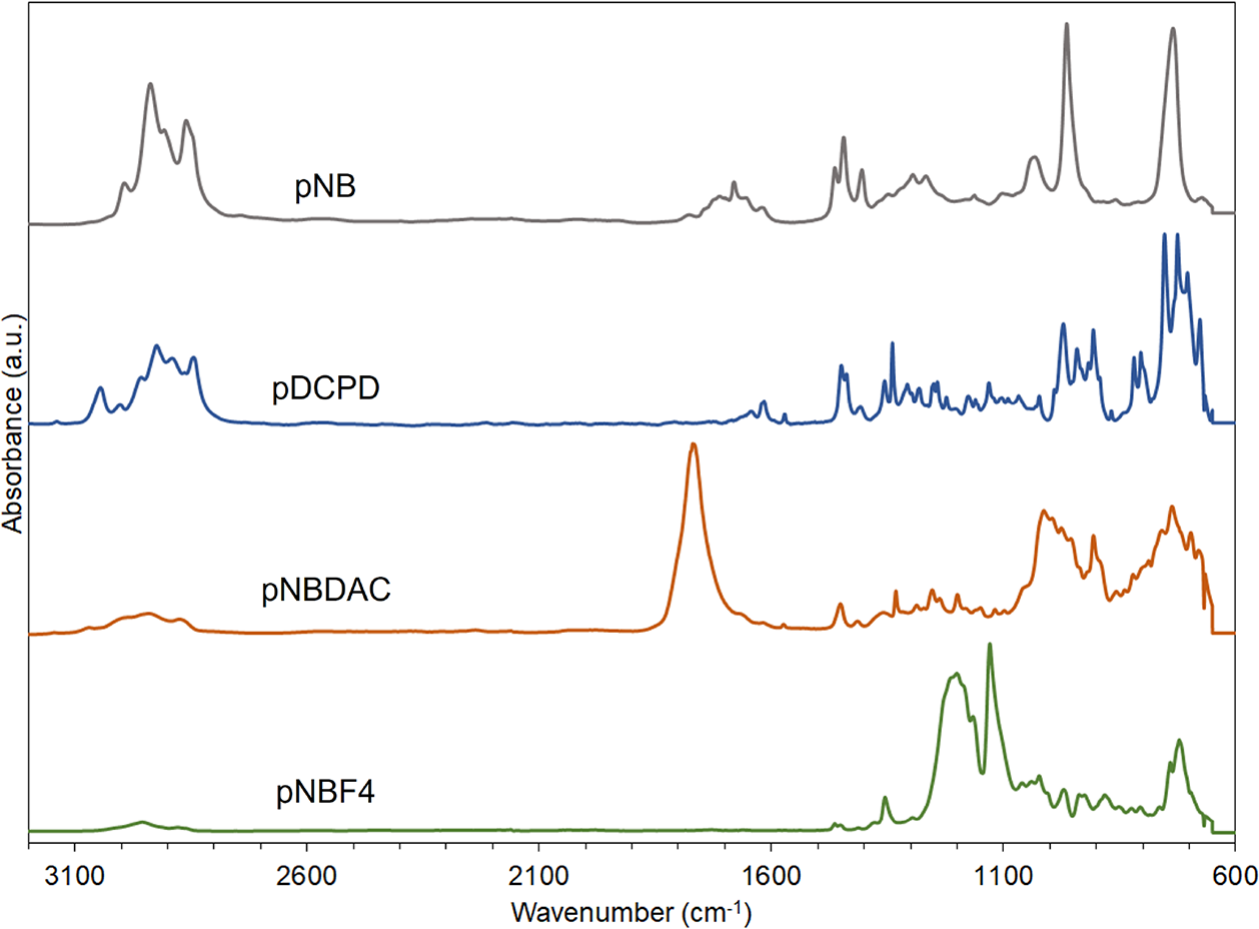
ATR-FTIR spectra of polymers made by using the scROMP process. From top to bottom: pNB; pDCPD; pNBDAC; and pNBF4. Films were made with a polymerization spin speed of 2000 rpm. Monomer concentrations were: NB, 4 M in DCM; DCPD, 2 M in DCM; NBDAC, 6.2 M (neat); and NBF4, 4.5 M (neat).

ATR-FTIR was utilized to confirm the synthesis of polymer films through the scROMP approach, as shown in Figure 2. Notably, the ATR-FTIR spectra of all polymer films synthesized exhibit peaks that are characteristic of typical polymers made by ROMP. A strong peak at 968 cm^–1^ corresponds to *trans* C=CH out-of-plane bending due to the olefin backbone, a doublet at 1451 and 1463 cm^–1^ is attributed to CH_2_ scissoring, peaks at 2936 and 2857 cm^–1^ correspond with acyclic CH_2_ stretching, a shoulder at 3010 cm^–1^ is identified as cyclic CH_2_ stretching, and a peak at 2910 cm^–1^ is attributed to C–H stretching. Other peaks present in the spectra indicate the functional groups present in the different polymers synthesized through the scROMP approach. In pNB and pDCPD, a distinct peak at 1447 cm^–1^ is attributed to the C–H bending. Additionally, pDCPD shows numerous distinct peaks in the C=CH out-of-plane bending region. This is attributed to the self-cross-linking nature of ROMP-synthesized DCPD.^29^ Films of pNBDAC show a strong peak at 1795 cm^–1^, which is indicative of the two pendant acyl chloride side chains from the repeat unit. Films of pNBF4 exhibit strong peaks at 1194 and 1126 cm^–1^ that are both attributed to C–F stretching along the perfluoro side chain.

### SEM Micrographs of scROMP Polymer Films

**Figure 3:**
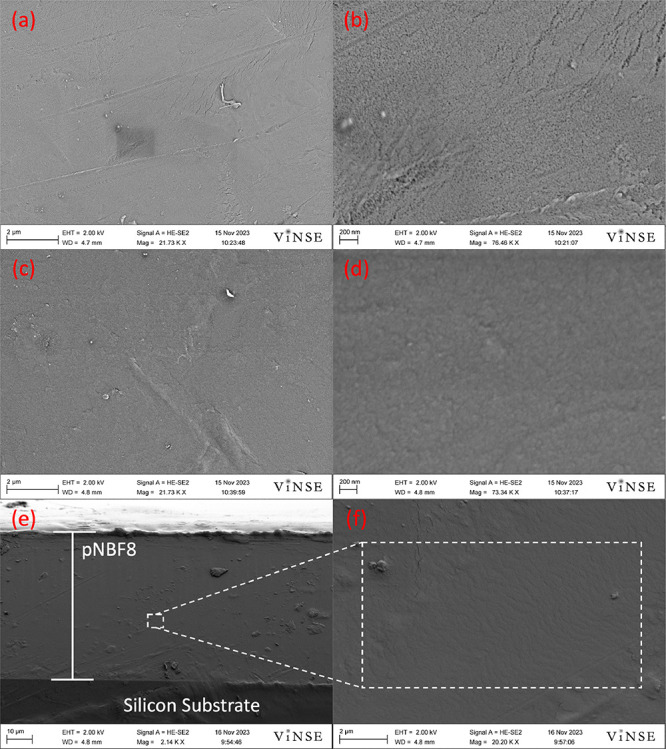
SEM micrographs of PAN and pNBF8 thin films: (a) top surface of PAN at 21K x magnification, (b) top surface of PAN at 76K× magnification, (c) top surface of pNBF8 on PAN at 21K× magnification, (d) top surface of pNBF8 on PAN at 73K× magnification, (e) cross section of pNBF8 on Au at 2K× magnification, and (f) Cross section of pNBF8 on Au-coated silicon at 20K× magnification.

SEM imaging of thin polymer films can reveal qualitative information such as porosity, surface coverage, and film uniformity. To investigate scROMP polymer films, a pNBF8 film was fabricated on a 4 cm^2^ support, either PAN (30 kDa MW cutoff) or Au at 1000 rpm. Synthesized pNBF8 films were fabricated on PAN to investigate surface and pore coverage and on Au-coated silicon substrates to image the cross section, as shown in Figure 3.

In Figure 3a,b, the top surface of a pristine PAN coupon shows visible pores on the surface, which is expected due to its commercial use as a support for nanofiltration purposes and its molecular weight cut off of 30 kDa. Following scROMP, Figure 3c,d shows the top surface of the pNBF8 film atop the PAN coupon. Importantly, the pNBF8 film surface shows that no pores are visible on the surface. The absence of visible pores on the surface of the pNBF8 film demonstrates that the scROMP process produces a polymer film that successfully covers the pores of the underlying support layer. Figure 3e shows the cross section of a pNBF8 film synthesized on a Au substrate, which indicates that the pNBF8 cross section is uniform with a film thickness of 40 μm at this low spin speed of 1000 rpm. Figure 3f is a close-up view of Figure 3e, revealing a dense polymer structure. The combination of these images demonstrates that the scROMP process can be utilized to synthesize a dense, nonporous polymer film atop both porous and nonporous substrates.

### Polymer Film Thickness

**Figure 4:**
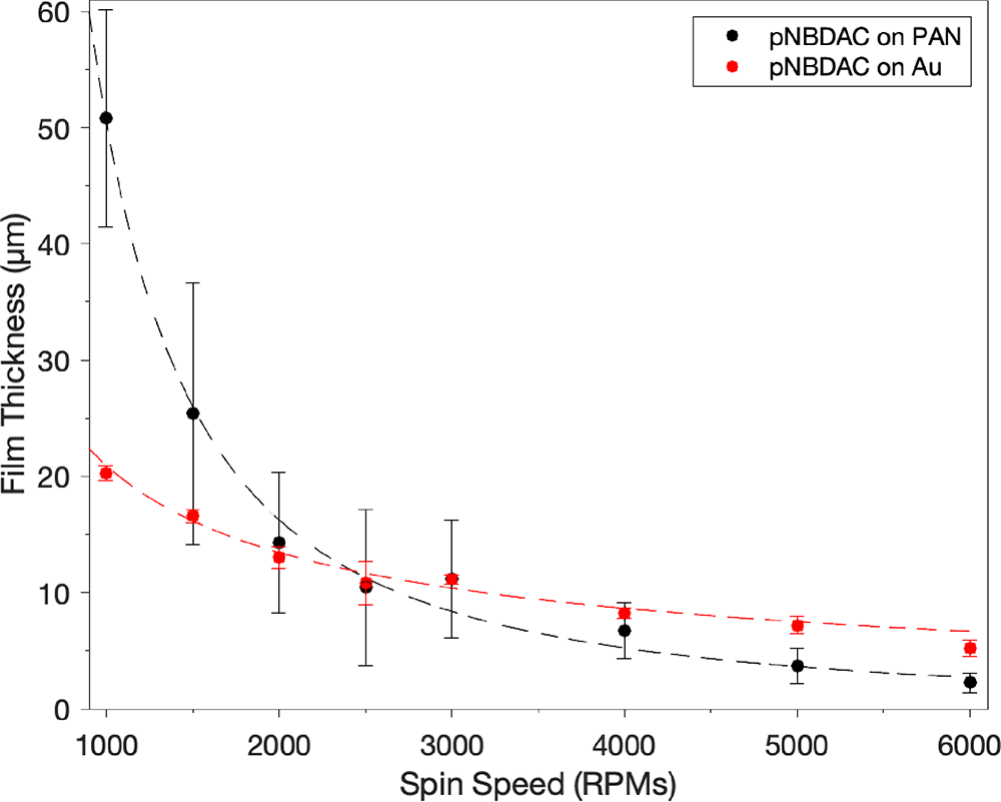
Profilometric thickness of films made by scROMP of neat NBDAC on a Au substrate (red) and a PAN support (black). Points represent an average of *n* > 3, and error bars represent the standard deviation between samples.

To quantify the impact that polymerization spin speed and substrate selection have on polymer film thickness, films were fabricated using neat NBDAC monomer on Au-coated Si/SiO_2_ wafers and on PAN at spin speeds ranging from 1000 to 6000 rpm, as shown in Figure 4. Profilometry was used to quantify film thickness, with an example profile provided in Figure S.3.

Thickness decreases with increasing spin speed on both substrates, albeit with a stronger dependence on PAN. Quantifying this effect of spin speed on thickness can be accomplished by analyzing how film thickness decreases proportionally to the spin speed (ω) as given by
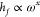
1

Employing this proportion on the thickness data reveals that pNBDAC films synthesized on Au-coated wafers exhibit a dependence of ω^–0.6^, which is comparable to values reported for the spin coating of polymer solutions onto surfaces with low roughness.^30,31^ Films synthesized on PAN exhibit a much stronger dependence of ω^–1.6^, which we attribute to the differences in porosity and roughness of the two surfaces (Figure S.4). The Au-coated Si/SiO_2_ wafer is a nonporous surface with low roughness (Ra = ∼3 nm), whereas PAN is a porous surface with much greater roughness (Ra = ∼3 μm).

The general trend of thickness with spin speed may be explained by considering momentum transfer at the surface, where the shear stress at the monomer−solid interface (τ) is described as
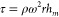
2where *r* is the radial coordinate, ρ is the monomer density and *h_m_* is the thickness of the unreacted monomer layer. As spin speed increases, the shear stress increases with a squared dependence, meaning that liquid spun on at twice the angular velocity will experience 4× the shear at the surface. This explains the continual thinning of polymer films grown at higher spin speeds on both surfaces, as the increased shear rate results in faster spin off of the monomer and unstabilized, small oligomers. Additionally, we investigated the impact of monomer volume dispensed on film thickness using neat liquid NBDAC and concluded that halving the monomer volume does not impact final film thickness significantly (Figure S.5).

**Figure 5:**
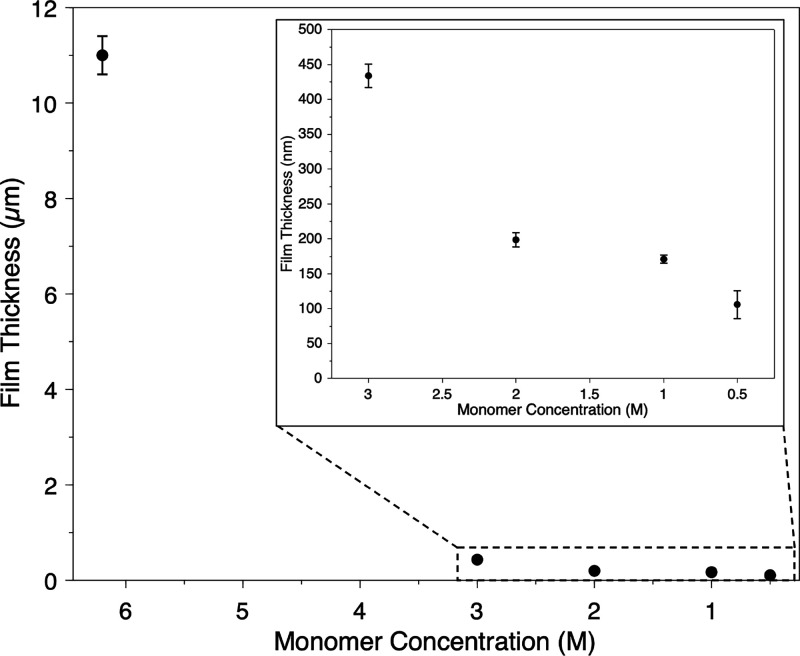
Effect of NBDAC monomer concentration on the resulting pNBDAC film thickness by scROMP at 3000 rpm. Monomer concentration of 6.2 M represents the neat liquid monomer. Monomer was diluted to lower concentrations in DCM. The inset enables clearer view of the film thicknesses and error bars at the reduced monomer concentrations.

While Figure 4 shows that film thickness can be varied from ∼50 down to a few μm by increasing spin speed with the neat NBDAC monomer, the concentration of the monomer solution dispensed atop the catalyst-laden surface will impact the resulting film thickness. The monomer NBDAC was used to study the influence that monomer concentration has on the resulting polymer film thickness, as shown in Figure 5. Following deposition of G3 onto a Au-coated substrate at 1000 rpm, solutions with different concentrations of NBDAC—6.2 (neat liquid monomer), 3, 2, 1, and 0.5 M in DCM—were dispensed onto the surface at 3000 rpm for 60s. As shown in Figure 5, scROMP of the neat liquid monomer at 3000 rpm results in a pNBDAC film that is 11 μm thick. Upon halving the monomer concentration, scROMP of 3 M NBDAC in DCM results in a film that is 25× thinner at 430 nm. The dramatic reduction in thickness upon the addition of a favorable solvent for both G3 and NBDAC shows the effects of catalyst and monomer spin-off on the process. Upon further dilution, the film thickness can be reduced to as thin as 100 nm. This study shows that scROMP can be utilized to generate submicrometer-thick films by varying monomer concentration. The approach of diluting the monomer concentration to generate thinner films has also been applied successfully to porous substrates.

### Modeling of the scROMP Process

**Scheme 2:**
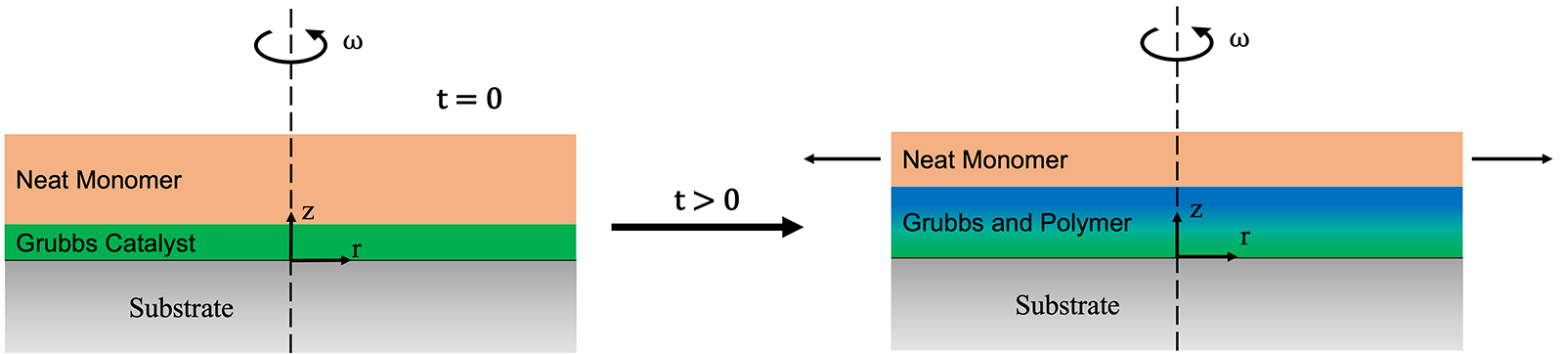
Schematic to Describe How the scROMP Process Is Modeled Using a Neat Monomer

To further our understanding of the scROMP process, we developed a model, shown in Scheme 2. For simplicity, this model approximates that at *t* = 0, a layer of neat monomer is interfaced with a layer of G3, and as the substrate begins to spin, the polymerization begins. This situation results in the (1) liquid monomer being spun off due to centrifugal force in the radial direction and (2) growth of the polymer layer in the *z*-direction due to polymer chain propagation. Based on the conservation of mass, the thickness of the unreacted monomer layer (*h*_m_) on the substrate is expected to scale as^32^
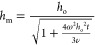
3where *h*_*o*_ is the initial film height, ω is the spin speed, *t* is the spin time, and υ is the kinematic viscosity of the fluid. This equation applies to a wide range of fluids used in spin coating and can be used to approximate the thickness of the remaining fluid on the substrate surface. The reaction of the monomer and catalyst is assumed to occur primarily at the interface between the layer of unreacted monomer and the layer of catalyst plus any grown polymer. As such, it is modeled as a surface reaction (*R*_S_):
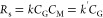
4where *k* is the reaction rate constant (cm^2^/mol-s), *C*_M_ is the areal concentration of neat monomer (mol/cm^2^), which is assumed constant while the liquid monomer is present, *C*_G_ is the areal concentration of active (initiated) G3 (mol/cm^2^), and *k*′ is the apparent rate constant (s^–1^). Equation 4 assumes that the initiation of G3 is rapid and, as such, does not consider initiation as a separate reaction step. Then, the velocity of the resulting polymerization reaction front is given as
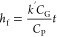
5where *h*_f_ is the polymer film thickness and *C*_P_ is the local concentration of polymer (mol/cm^2^). Utilizing these three equations allows for the interplay between convective monomer transfer and polymerization front velocity within the scROMP process to be studied.

**Figure 6:**
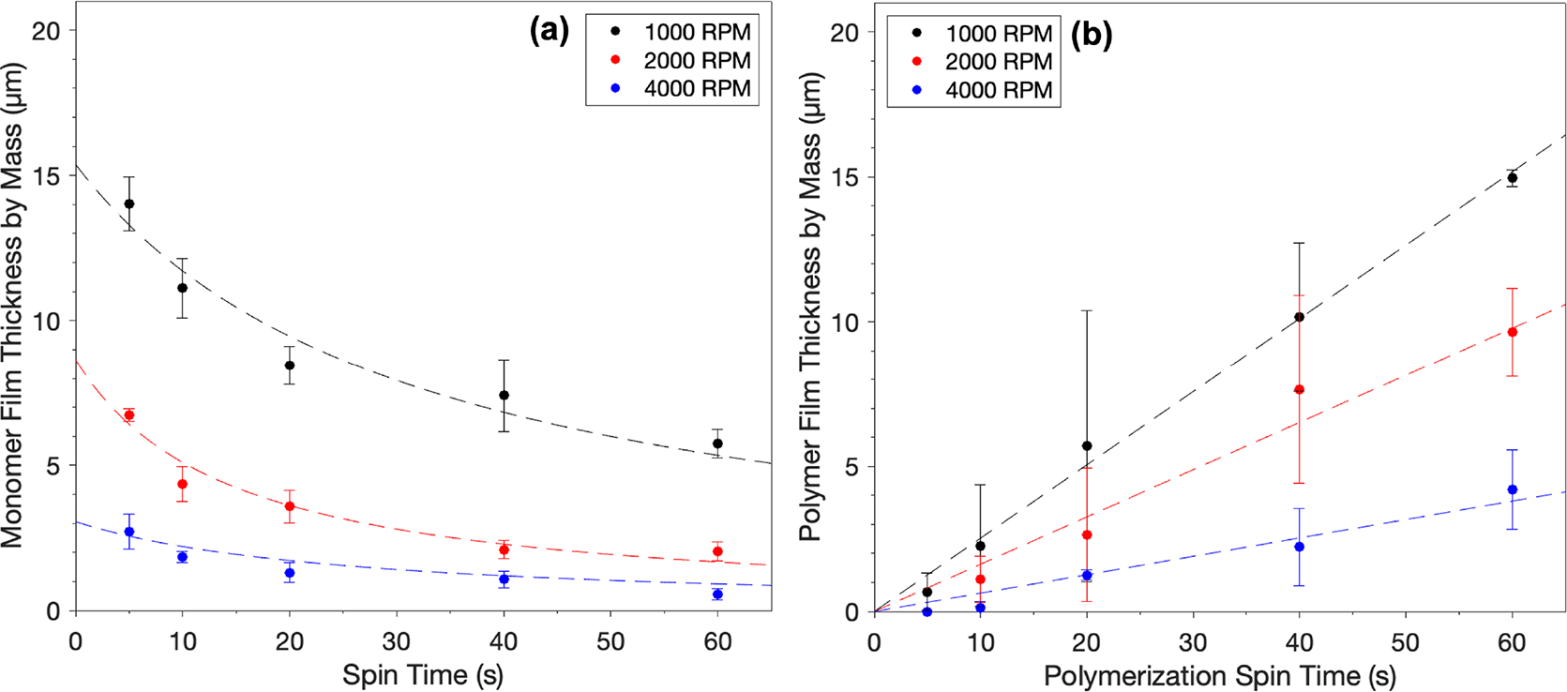
Monomer layer thickness (a) and polymer film thickness (b) as a function of spin time. Neat NBDAC monomer was applied to a spinning surface without catalyst in (a) and with catalyst in (b). In (b), polymerization was stopped at the indicated times by spinning on ethyl vinyl ether as a ROMP termination agent. Data points represent the mean, and error bars represent the standard deviation of at least three independent samples. Dashed curves represent fits of the data with eq 3 in (a) and eq 5 in (b).

To determine this balance between monomer spin off and polymerization, we performed a set of experiments in which monomer was spun onto surfaces with and without a catalyst as shown in Figure 6. Following spinning, the change in the mass (Δ*m*) of the substrate was determined. The average thickness of monomer (*h*_m_) on the substrate was then determined from the mass change as
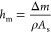
6where ρ is density of the monomer NBDAC (ρ = 1.349 g/cm^3^) and *A*_S_ is the substrate surface area.

Figure 6a shows how the thickness of the NBDAC monomer layer decreases over time when spun onto a surface without a catalyst. The centrifugal force imparted by the spinning substrate results in the radial convective transfer of the monomer from the surface and the thinning of the fluid film, as modeled well by eq 3.

Figure 6b shows polymer growth over time when the monomer is spun onto a catalyst-loaded substrate. After the allotted time, the polymerization was stopped by spinning on ethyl vinyl ether, a termination agent. The polymer thickness increases with spinning time at all spin speeds and decreases with increasing spin speed, as greater spin speeds increase the rate of convective monomer spin off and may also cause spin off of the catalyst and newly formed oligomers. The increase in the polymer film thickness with time is linear for all spin speeds analyzed. Based on eq 5, this linear dependence indicates that the concentration of active G3 is approximately constant during the spinning at a specific speed and that unreacted monomer is available to fuel the reaction throughout the 60 s of spinning. Thus, monomer units continue to add to the polymer chain until all monomer at the interface is consumed or the catalyst is deactivated, whichever comes first.

Comparing the data in Figure 6a,b reveals a key observation about the scROMP process. Polymer thicknesses at 1000 and 4000 rpm are 6× and 3× greater than the thicknesses of the respective monomer layers at 60 s. This result illustrates the impact of G3 being present on the surface. As G3 initiates the polymerization and polymer chain growth begins, the rate of monomer spin-off is decreased. This decrease in monomer loss is due to the thinning of the unreacted monomer film caused by polymer growth along with the likelihood that growing polymer chains impede monomer spin off by entraining the monomer. Consequently, the growing polymer film retains more monomer on the surface, which is subsequently incorporated into the propagating polymer film.

### Polymer Properties and Polymerization Kinetics of scROMP

ROMP with G3 is known for producing polymers with easily tunable molecular weights and low PDIs.^14,22,23,33^ The scROMP process utilizes G3 due to its rapid initiation rate in solution polymerizations (*k* > 4 s^–1^),^14^ its tolerance to ambient conditions, and its production of polymers with low PDI (*Đ* ≤ 1.3).^22,33^ These published values are reported for polymerizations that were carried out in solution, often under stringent reaction conditions. As the scROMP approach is carried out at ambient conditions, namely, in a fume hood with convective air flow, we sought to determine the effect of the scROMP process on the *M*_W_ and PDI of the synthesized polymers. To accomplish this, pNBDAC was made by conducting scROMP at 1000, 2000, and 4000 rpm using the neat monomer NBDAC, exactly as described above for the results of Figure 5. After 60 s, the surface was flooded with ethyl vinyl ether, terminating the polymerization reaction. Following synthesis of pNBDAC, the film was immersed in ethanol to convert the acyl chloride side chains into ethyl ester-terminated side chains. The resulting polymer is soluble in THF and can be analyzed by GPC/SEC to determine the *M*_W_ and PDI, which are shown in Table 1.

**Table 1:** Polymer *M*_W_ and PDI for the scROMP of NBDAC at Varying Spin Speeds

spin speed (RPM)	pNBDAC *M*_W_ (kDa)	pNBDAC PDI (*Đ*)
1000	404.6	1.17
2000	480.6	1.13
4000	635.9	1.13

The results in Table 1 show that pNBDAC synthesized through scROMP exhibits high *M*_W_ (>400 kDa) and low PDI (<1.2), which are attributed to the fast initiation and propagation rates of ROMP using G3, as well as the high concentration of monomer (neat NBDAC ≈ 6.2 M). As the spin speed increases, the polymer molecular weight continually increases and polydispersity decreases. The combination of higher molecular weights (Table 1) and lower film thicknesses (Figure 6b) at the higher spin speeds is consistent with a reduced utilization of active catalyst due to the much higher shear stresses present at the monomer/catalyst interface at higher spin speeds (eq 2). Since the catalyst is not chemically anchored to the surface, the addition of liquid monomer to the surface at such high shear stress can result in the catalyst and lower molecular weight oligomers being swept off the surface during convective monomer spin off. Nonetheless, the high initiation and propagation rates of G3 still cause sufficient polymer growth at the monomer–catalyst interface. The decrease in active G3 concentration at higher spin speeds means there are fewer catalysts competing to initiate and subsequently propagate chain growth; hence, the higher *M*_W_ and lower PDI with increasing spin speed. An additional study was performed to evaluate the impact that the G3 amount had on *M*_W_ and PDI of pNB synthesized by performing scROMP of 1 M NB in DCM as shown in Table S.1.

Data from Figure 6a,b and Table 1 can be used in conjunction with eqs 3–5 to extract key parameters regarding the behavior of polymer growth during the scROMP process. The *M*_W_ of pNBDAC films and the mass of the synthesized film were used to calculate the amount of active G3 during scROMP at the analyzed spin speeds (Table S.2), which does indeed decrease with spin speed. Knowing the amount of G3 actively contributing to polymer growth allows for the apparent rate constant, *k*′, to be determined by the slope of the linear fit in Figure 6b. Assuming that *C*_P_, *C*_M_, and *C*_G_ are all constant throughout the polymerization process, eqs 4 and 5 can be rearranged to determine *k*′, *k*, and *R*_S_ as shown in Table 2.

**Table 2:** Kinetic Parameters for scROMP of Neat NBDAC at Varying Spin Speeds

spin speed	apparent rate constant, *k*′	active G3 concentration, *C*_G_	rate constant, *k*	surface reaction rate, *R*_S_
(RPM)	(s^–1^)	(mol/cm^2^)	(cm^2^/mol-s)	(mol/cm^2^-s)
1000	40	3.8 × 10^–9^	1.3 × 10^5^	15 × 10^–8^
2000	49	2.0 × 10^–9^	1.6 × 10^5^	9.6 × 10^–8^
4000	63	0.6 × 10^–9^	2.1 × 10^5^	3.8 × 10^–8^

Values of *k*′ represent the frequency in which monomer units are added to the propagating polymer chain, which are comparable at different spin speeds. Using the determined *C*_G_ and *k*′ values, the values of *k* and *R*_S_ can be determined. Values of *R*_S_ illustrate that the rate of polymer growth decreases with an increase in spin speed, which is verified by the growth of thicker films at lower spin speeds over the same time frame. Notably, the rate of film growth observed for the scROMP approach is much higher than those previously observed by our group using different siROMP approaches.^28,34,35^ We attribute the higher rate of film growth to the fact that G3 is not bound to the substrate. As the monomer is dispensed on the surface, a mobile G3 can more effectively orient itself into a favorable position for propagation. This results in films 6×–20× thicker than those made with only bound catalyst using similar surface- and macro-initiated approaches to film synthesis, as reported by Escobar et al. for similar reaction times.^34^

### Random Copolymers from scROMP

An important feature of scROMP is its ability to simultaneously polymerize different monomers to form random copolymer films. We have combined targeted comonomer chemistries using scROMP to generate copolymer films that possess either hybrid or novel properties as compared to those of their respective homopolymers. The combination of NB with a semifluorinated monomer such as NBF4 can be used to investigate the effect of fluorocarbon fraction on film surface properties.

**Figure 7:**
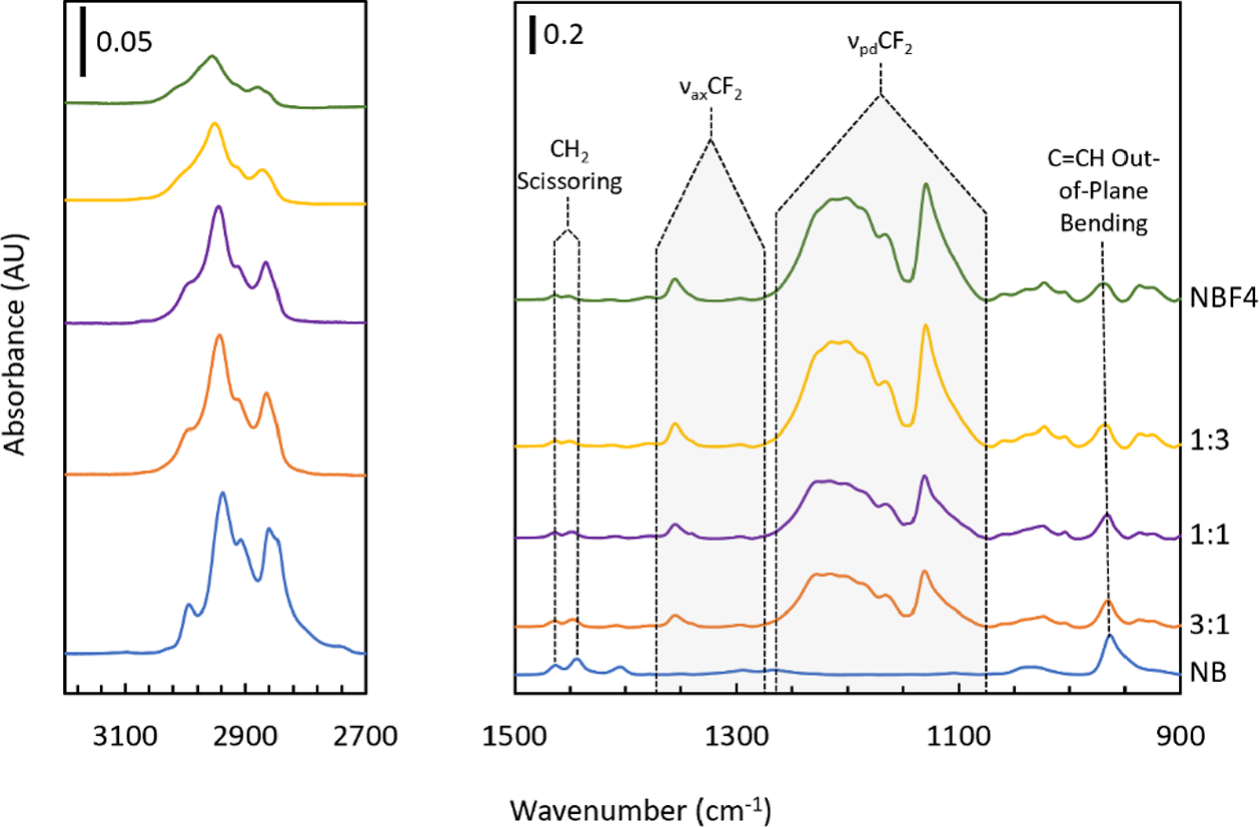
ATR-FTIR spectra of p(NB-*co*-NBF4) films with monomer ratios listed as molar ratios of NB to NBF4. The spectra on the left are due to C–H stretching, and the important functionalities on the right are indicated in the plot.

Figure 7 shows ATR-IR spectra of the homopolymer films pNB and pNBF4 along with p(NB-*co*-NBF4) films at NB:NBF4 reagent ratios of 1:3, 1:1, and 3:1 with monomer solution concentrations totaling 4.5 M in pentane. The films reported in Figure 7 ranged from 3.2 to 8.2 μm in thickness. The hydrocarbon stretching region (3050–2750 cm^–1^), CH_2_ scissoring peaks, and C=CH out-of-plane bending peak are all more prominent in films with a higher pNB concentration. In contrast, the axial and perpendicular fluorocarbon stretching regions increase with increasing pNBF4 concentration. These IR spectra suggest that the compositions of the copolymer film can be varied by changing the ratio of the two reagents, and GPC/SEC data in Table S.3 show that solubility in THF is impacted by varying monomer solution ratios.

More precise molar percentages of the copolymer films were assessed by using ^1^H NMR (see Figures S.6–S.8) and reported in Table 3. Additionally, HSQC was performed on the pNBF4 film for structural analysis, as reported in Figure S.9. From Table 3, NBF4 is preferentially incorporated into the copolymer films when compared to each respective monomer ratio. This enrichment in the fluorocarbon component of the copolymer is attributed to enhanced partitioning of the NBF4 monomer to the catalyst in increasingly fluorocarbon regions.

**Table 3:** Molar Percentage of pNBF4 for Various Monomer Ratios Based On ^1^H NMR; Water and Hexadecane Contact Angles Correspond to Films Made Using the Same Monomer Solution Ratios

		water contact angle		hexadecane contact angle	
monomer ratio	% pNBF4	adv.	rec.	adv.	rec.
NBF4	100	113 ± 1	94 ± 2	50 ± 1	<20
1:3	86	113 ± 1	96 ± 2	52 ± 2	<20
1:1	64	111 ± 1	94 ± 1	54 ± 2	<20
3:1	30	111 ± 1	95 ± 1	54 ± 2	<20
NB	0	94 ± 1	76 ± 2	24 ± 1	<20

Whereas ^1^H NMR is useful to assess the bulk composition of the films, wetting measurements or contact angles are sensitive to the outermost 0.3–0.5 nm of the surface.^36^ As probe liquids for contact angles, we have used water and hexadecane for their ability to distinguish −CF_3_ and −CF_2_– surface compositions that are likely present for pNBF4 from −CH_2_– compositions that dominate for pNB. In a comparison of the homopolymer films, pNBF4 exhibits water and hexadecane contact angles that are 19 and 26° higher than those of pNB, reflecting the presence of fluorocarbon groups on the pNBF4 surface. pNBF4 advancing contact angles of 113 and 50° for water and hexadecane, respectively, are comparable to values of 110 and 61° reported for the pNBF4 films by Faulkner et al.^26^ using siROMP. The increase in water contact angle and decrease in hexadecane contact angle here attributed to greater roughness for the thicker polymer film formed via scROMP. Remarkably, the water and hexadecane contact angles for all copolymer films are approximately the same as those for pNBF4. These constant contact angles with decreasing monomer ratio indicate that the polymer chains must rapidly adjust to place the low-energy perfluoro chains at the surface, dominating the surface even at low concentrations in the bulk. This reorientation of fluorocarbon groups at the surface to minimize interfacial free energy is consistent with some other semifluorinated films,^37−40^ as the repulsion of the fluorocarbon side chains by the hydrocarbon backbone creates localized ordering of the fluorocarbon chains. This rearrangement to form the dense fluorocarbon surface layer may prove useful as an antiswelling, shielding layer for membranes, where the rapid flux of a liquid under separation can cause significant swelling of the polymer and hamper the separation.^41^

**Figure 8:**
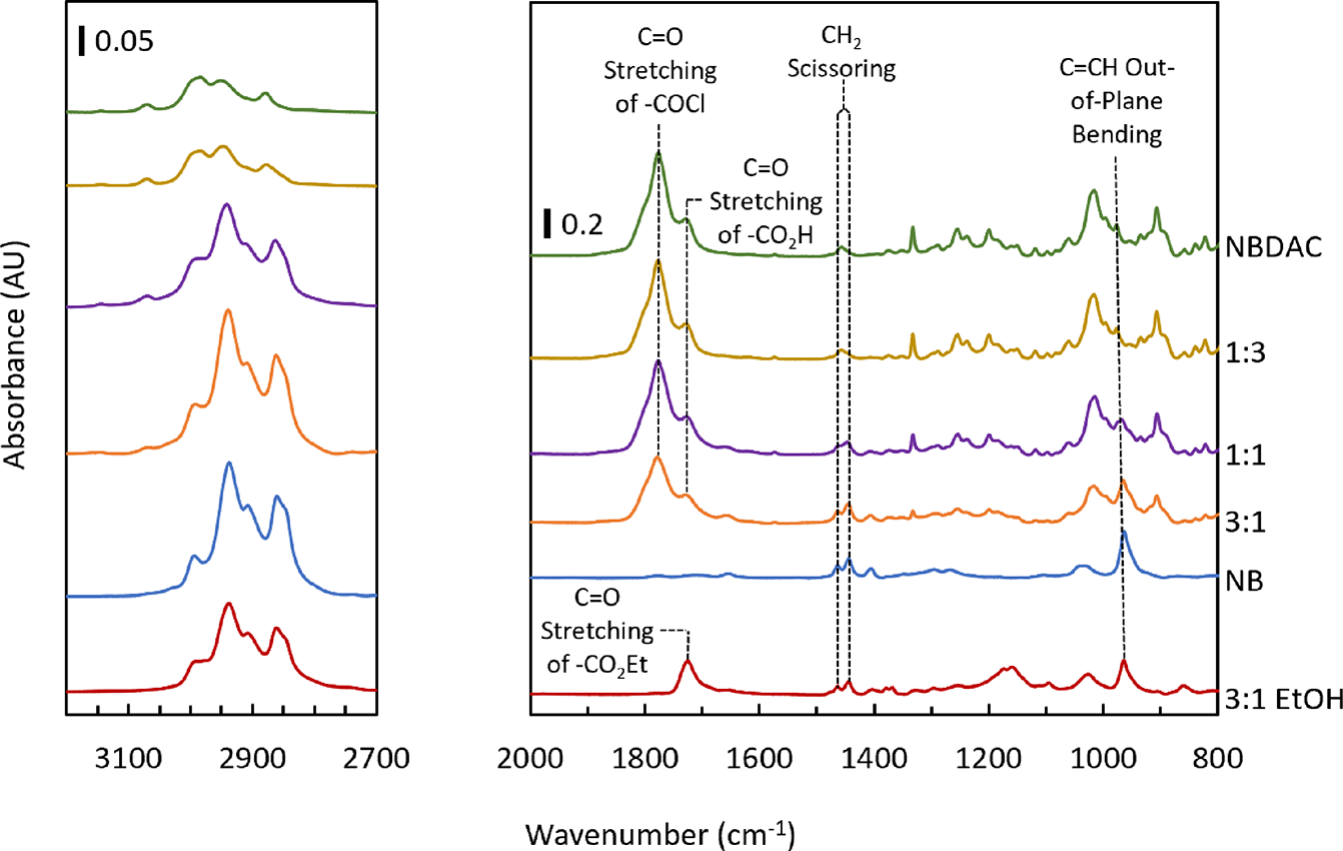
ATR-FTIR spectra of p(NB-co-NBDAC) films with monomer ratios listed as molar ratios of NB to NBDAC. The bottom spectrum is the 3:1 film after 1 h of immersion in ethanol.

Another scROMP copolymer system of interest is p(NB-co-NBDAC), particularly for the acyl chloride functionality of NBDAC that can be readily modified after polymerization to introduce controlled functionality into a nonpolar polymer matrix.^28^ Films of p(NB-co-NBDAC) were synthesized by scROMP of varying monomer ratios totaling 4 M in pentane. In Figure 8, the hydrocarbon stretching region (3050–2750 cm^–1^), CH_2_ scissoring peaks, and C=CH out-of-plane bending peak are again more prominent in films with higher pNB concentration. The C=O stretching of the acyl chloride is more prominent in films with greater pNBDAC concentrations. Since the acyl chloride will readily react with moisture in the atmosphere, some of the C=O stretching peak is shifted to the C=O stretching of the carboxylic acid after exposure of the film to air for a few minutes. To mitigate this, fresh p(NB-co-NBDAC) films can be immersed in ethanol for 1 h to fully convert the acyl chloride to the ethyl ester (Figure 7), which is both stable and forms at ∼60× the rate of the carboxylic acid upon exposure to aqueous ethanol solutions.^42^ This conversion highlights the versatility of p(NB-co-NBDAC) films as the acyl chloride will react with most alcohols or amines, given sufficient immersion time. A multitude of copolymer films can then be synthesized from this starting scaffold, creating copolymers with carboxylic acid, ester, or amide functionality.

### Using the scROMP Approach to Fabricate Thin-Film Composite Membranes

The scROMP approach can rapidly fabricate thin, uniform polymer films on a variety of substrates. To further demonstrate the versatility of the scROMP process, films were fabricated on PAN coupons with a 30 kDa Mw cutoff and utilized to dehydrate ethanol. The dehydration of polar solvents is a particularly important separation.^43,44^ Current thermal-based separation processes account for 12–16% of U.S. energy consumption per year.^43^ Distillation alone accounts for 50% of that range, incentivizing alternative separation methods to be investigated. Membrane pervaporation is quickly gaining traction as an alternative to distillation due to its relatively low energy consumption, lower capital costs, and ability to circumvent azeotropes in aqueous polar solvent mixtures.^45,46^

Thin film composite membranes (TFC) were fabricated with the monomers shown previously and used to dehydrate ethanol. For this purpose, films were scaled from a substrate size of 4 to 36 cm^2^ to ensure the membrane’s surface fully covers the testing cell’s inlet. This scale up was done by increasing the volume of catalyst solution and monomer dispensed on the surface. Substrates at the 4 cm^2^ size scale had 200 μL of G3 solution and 200 μL of monomer dispensed on the surface. For the 36 cm^2^ size scale, these solution volumes were increased to 400 μL of the G3 solution and 400 μL of the monomer. We observed that increasing the surface area 9× required only a 2× increase in dispensed solution volumes to achieve a uniform film on the surface of the PAN support. If the solution dispense volume is insufficient for the catalyst or the monomer, the surface will not be uniformly coated. The 9× increase in surface area requiring only a 2× increase in catalyst and monomer volumes is a benefit of the scROMP process, as the use of solvent and high-value components can be minimized while still forming a uniform polymer film.

We investigated the dehydration of ethanol using a few different TFC compositions. The scROMP process enables the rapid fabrication of many TFC membranes with different selective layer compositions. In this study, we synthesized films of pNB and pNBF6, as well as pNBDAC that was subsequently modified postpolymerization with various primary amines and alcohols to impart additional hydrophilic or hydrophobic functionality with two side chains per repeat. As shown previously in our group,^28^ pNBDAC arises as an intriguing polymer due to the two acyl chloride pendant groups on each repeat unit. These pendant groups can be modified postpolymerization to a vast number of functional moieties simply through exposure to amines or alcohols. The synthesized TFC membranes were then exposed to a recirculating 90/10 (v/v)% EtOH/H_2_O solution heated to 55 °C. A vacuum pump equipped on the permeate side of the membrane cell induced a pressure differential of ∼−99.5 kPa to promote vapor permeation. The permeating vapor was then condensed in a cold trap cooled by liquid nitrogen (Scheme S.1 shows a schematic of this setup). The separation performances of these films are shown in Table 4, denoting the side chain that emanates from the polynorbornene backbone. Membrane performance is governed by
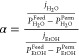
7where *j*_i_ is component molar flux (mol/m^2^ h), *P*_i_^Feed^ is the component vapor pressure in the feed stream (mmHg), and *P*_i_^Perm^ is the component vapor pressure in the permeate stream (mmHg). The membrane selectivity, α, indicates how well a membrane separates component *i* from another component, where a value of α > 1 indicates component *i* is concentrated in the permeate stream. For our system, α > 1 shows an enrichment of water in the permeate stream.

**Table 4:** EtOH Dehydration Performance of TFC Made by scROMP that Contains Different Side Chains on a Polynorbornene Backbone

	total flux	H_2_O/EtOH selectivity	permeate EtOH content
TFC side chain^a^	(g/m^2^-h)	(α)	(wt %)
control, no TFC	34000	2.1	82
*p*-methoxyphenyl amide	3300	2.4	81
carboxylic acid	8100	2.6	80
amidoethanesulfonic acid	5700	3.5	75
pentafluorophenyl ester	8700	4.4	71
ethyl ester	8400	4.8	68
*o*-methoxyphenyl amide	200	7.9	58
perfluorohexyl	170	14.0	49
phenyl amide	1900	16.4	41

a Aside from perfluorohexyl, which has only one side chain per repeat, all other reported side chains have two side chains per repeat.

As shown as a control in Table 4, the pristine PAN support yields a low α of 2.1 and a large flux, showing that the film is slightly selective toward water but still permeates a solution that is comparable to that of the feed. The scROMP approach is capable of producing TFC membranes that are selective to water over ethanol. TFC formation is exhibited by all monomers, and all of these TFC membranes show a decrease in total flux and increase in selectivity as compared to the pristine PAN control. The improved membrane performance is due to the formation of a dense polymeric active layer from scROMP that increases mass transfer resistance and enables the smaller water molecules to permeate more rapidly than ethanol. Of the compositions shown in Table 4, the films possessing hydrophilic side chains, such as carboxylic acid and amidoethanesulfonic acid, show high fluxes and mild selectivities toward water over ethanol. The lower selectivities from the hydrophilic side chains can be attributed to high degrees of swelling, as these films have no cross-linker. Nonetheless, these films do show a preference for water over ethanol, suggesting that high-performance films with hydrophilic side chains can be generated through scROMP with appropriate cross-linking. Films with hydrophobic side chains, such as perfluorohexyl for pNBF6, have a much higher selectivity than the hydrophilic films, with the trade-off of a lower flux. Hydrophobic films with higher fluxes can be generated, as shown by a pNBDAC film modified by using aniline to generate phenyl amide side chains. This hydrophobic film yields a robust selectivity of 16 along with a flux that is 1 order of magnitude greater than that for pNBF6. This specific polymer composition and other similar pNBDAC modifications will be the focus of future work in which scROMP parameters will be tuned to improve separation performance. Thus, the scROMP approach enables the rapid fabrication of many different polymer TFC compositions to facilitate polymeric materials discovery for separation applications.

## Conclusions

The scROMP process combines spin coating with ROMP to produce a method that merges polymer synthesis and film deposition into a single rapid process. By utilizing a mobile catalyst, scROMP can surpass some of the constraints of surface-initiated or “grafting from” processes, including access to much thicker films and to removable films for more detailed polymer chain characterization. Our work has probed the capabilities of the scROMP approach such as its ability to accommodate a range of NB-type monomers, to produce random copolymer films, and to tune the polymer film thickness from 0.1 to 50 μm through adjusting spin speed and monomer concentration during the polymerization step. SEM images reveal that the scROMP technique produces a dense, nonporous polymer film that fully covers the surface atop both porous and nonporous substrates. Importantly, the polymers in the films produced by scROMP possess high *M*_W_ (>100 kDa) and low PDI (<1.2).

In combination with GPC/SEC data, the model developed here also allows for the extraction of key kinetic parameters of scROMP, showing that film growth is 1 order of magnitude more rapid than surface-initiated approaches. Additionally, high chain molecular weights are favored by faster spin speeds, where the higher centrifugal forces lead to the spin off of some catalyst and oligomers. Lastly, we demonstrate the ability to generate TFC membranes using scROMP. By using the monomer NBDAC, we can modify these TFC postpolymerization through amine or alcohol exposure to introduce additional functionality. This versatile synthesis generated a polymeric template that was used to investigate the polymer performance in dehydrating aqueous ethanol solutions by pervaporation.

## Supporting Information Available

Digital images of polymer films, stability analysis of polymer films, impact of monomer volume, calculations of active (initiated) G3 during polymerization, NMR analysis of copolymers, including HSQC, GPC/SEC data for additional homopolymers and copolymers, and membrane pervaporation equipment setup (PDF)
