# Supplementary material for: Simultaneous Spin Coating and Ring-Opening Metathesis Polymerization for the Rapid Synthesis of Polymer Films
Source: ACS Appl Mater Interfaces. 2024 Mar 22;16(13):16754–66. doi: 10.1021/acsami.4c00211 (PMC10995903; doi:10.1021/acsami.4c00211)
Supplement: Supplementary file 1 — am4c00211_si_001.pdf [file am4c00211_si_001.pdf]

Supporting Information

For

Simultaneous Spin Coating and Ring-Opening Metathesis Polymerization for the Rapid Synthesis of Polymer Films

Zane J. Parkerson[1], Liudmyla Prozorovska[2], Matthew P. Vasuta[2], Tyler D. Oddo[1], and G. Kane Jennings[1,]*

[1] Department of Chemical and Biomolecular Engineering, Vanderbilt University, Nashville, TN 37235

[2] Interdisciplinary Materials Science Program, Vanderbilt University, Nashville, TN, 37235

Corresponding Author: *G. Kane Jennings, Department of Chemical and Biomolecular Engineering, School of Engineering, Vanderbilt University, Nashville, TN, 37235; Email: kane.g.jennings@vanderbilt.edu

SI.1 Synthesis Routes for scROMP on Various Substrates

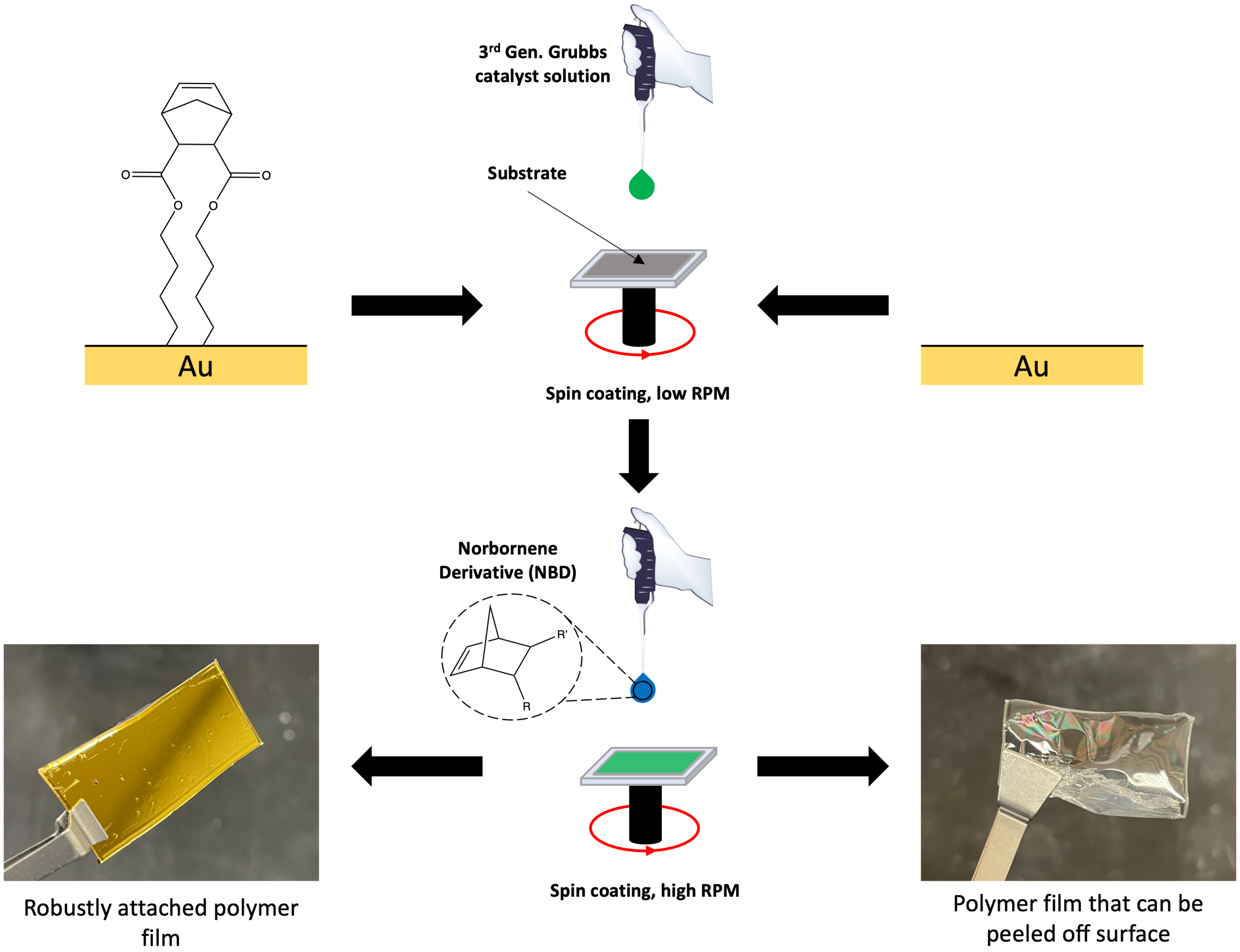

Figure S.1. Schematic depicting two pathways for synthesizing pNBD films. (Left) Synthesis of a pNBD film by scROMP atop a substrate possessing a norbornene-terminated SAM. (Right) Synthesis of a pNBD film by scROMP atop a pristine Au-coated wafer. The left pathway yields a robustly attached polymer film whereas the right pathway yields a film that can be peeled away, intact, from the surface.

SI.2 Polymer Film Stability Testing

Polymer film stability on PAN supports was evaluated by flowing a 90/10 v/v% EtOH heated to 55 ºC across a 4 cm^2 polymer film for 6 h, similar to the environment for the ethanol dehydration studies. Prior to flow testing, a pNBF8 film was analyzed with ATR-FTIR to establish a baseline absorbance. Following flow testing, the same film was reanalyzed with ATR-FTIR to observe the change in absorbance as shown in Figure S.2.

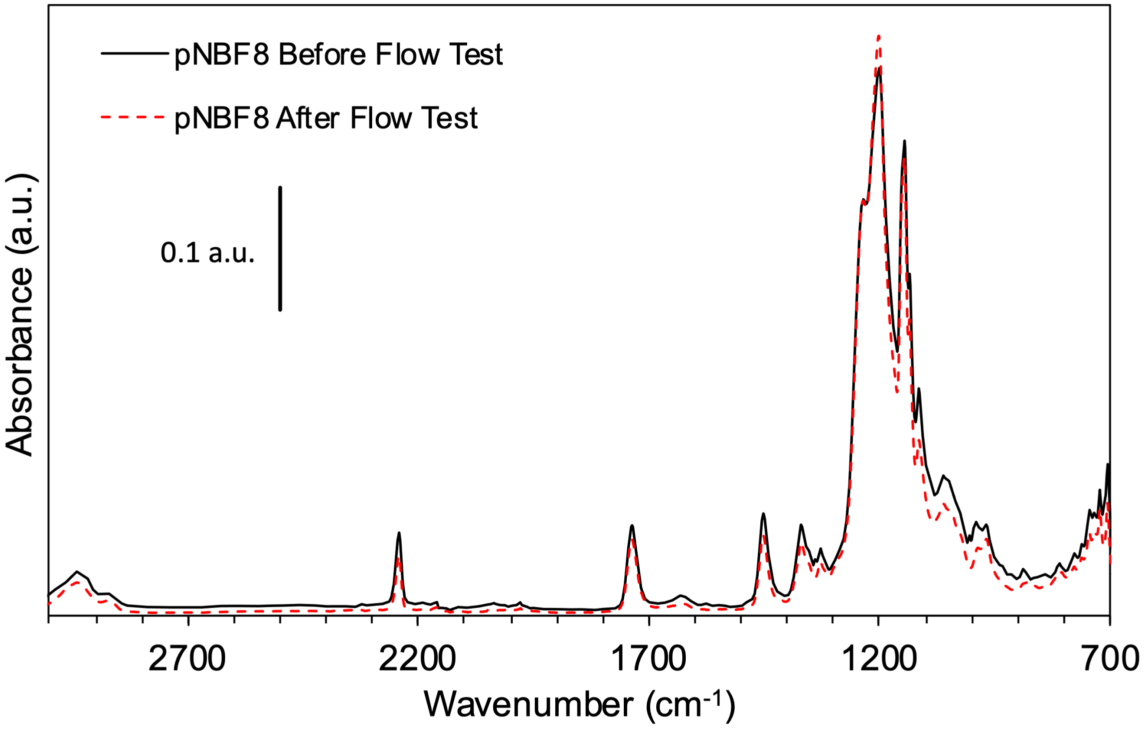

Figure S.2. ATR-FTIR absorbances of a pNBF8 film synthesized on PAN before (black, solid) and after (red, dashed) flow cell testing.

The pNBF8 film on PAN shows little to no change in ATR-FTIR absorbances for C-F stretching (1100- 1300 cm^{-1}) as well as signals corresponding to the underlying support (ester stretching at 1730 cm^{-1} and nitrile stretching at 2240 cm^{-1}) after testing in the heated ethanol and water mixture

for 6 h. If the film was being removed from the surface during the flow testing, even partially, we would expect to see a decrease in C-F stretching and increases in those signals due to the support. The similarity in these spectra indicates that the polymer film is robustly adhered to the PAN support and is not desorbed or eroded during membrane flow testing.

SI.3 Profilometric Measurements of Polymer Film on Au Substrate

Stylus profilometry was performed to quantify film thickness and surface roughness. Figure S.3 shows an example profilometric line scan of a polymer film on a Au-coated wafer.

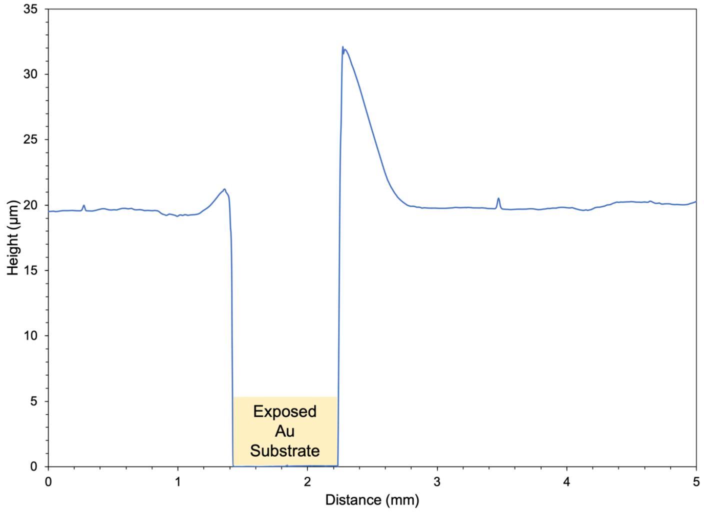

Figure S.3. Profilometric line scan of pNBDAC on Au by scROMP. The pNBDAC film was made by scROMP of neat liquid NBDAC monomer (6.2 M) at a polymerization spin speed of 1000 RPM.

Profilometry measurements were conducted after performing scROMP using neat NBDAC. Prior to profilometry, a portion of the pNBDAC film was removed to expose the Au substrate so that film height could be measured relative to the substrate. Over the sampled distance, the film shows a uniform thickness of 20 μm and a comparably low surface roughness (Ra = 85 nm).

SI.4 Surface Roughness of PAN and Au-Coated Wafer

The surface roughness of the PAN support and Au-coated wafer were quantified by stylus profilometry. Line scans across the surface of both substrates are shown below in Figure S.4.

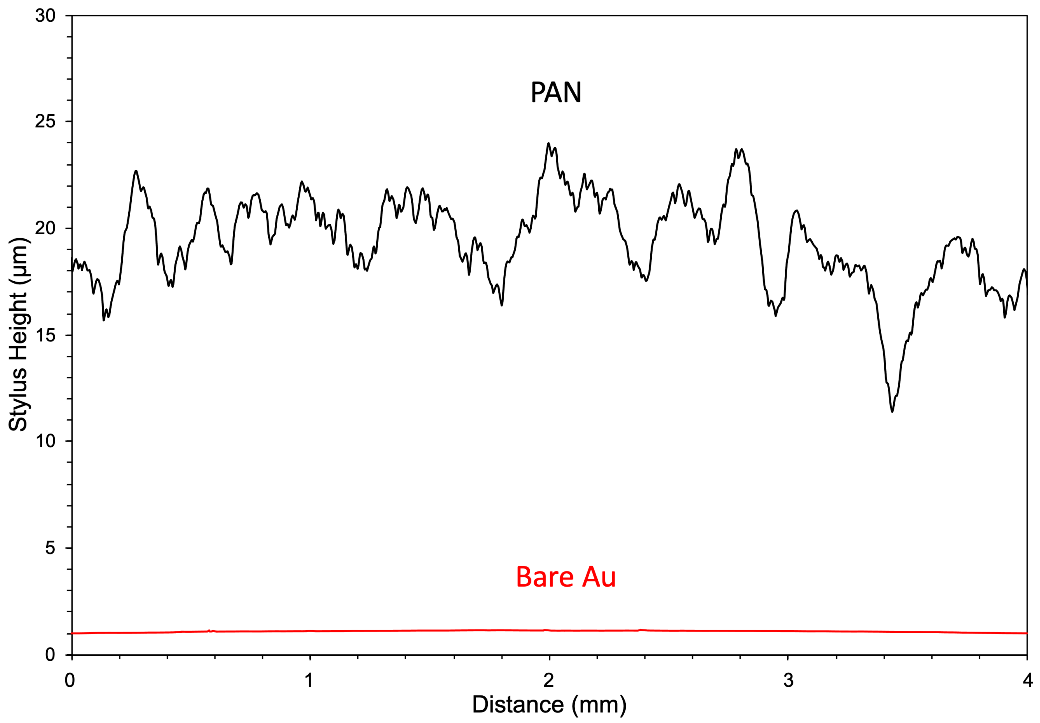

Figure S.4. Profilometric line scans of the surface of PAN (black) and a Au-coated wafer (red). Line scans are offset to provide better visualization of the roughness of the surfaces.

Profilometry of the pristine surfaces shows that the non-porous Au substrate has a very low surface roughness (Ra = ~3 nm) whereas the porous PAN surface has orders-of-magnitude higher surface roughness (Ra = ~3 µm). We attribute the higher dependence of ω observed on PAN to the increased surface roughness and presence of porosity.

SI.5 Impact of Monomer Dispense Volume on Film Thickness

The amount of monomer dispensed on the surface during the polymerization step of scROMP was assumed to impact the resulting film thickness. To investigate this, polymer films were made atop a 4 cm^2 Au-coated wafer at varying polymerization spin speeds using 100 and 200 μL of NBDAC as shown in Figure S.5. This study revealed that there is no significant difference between the final film thickness when either 100 μL or 200 μL of the neat liquid monomer NBDAC is used.

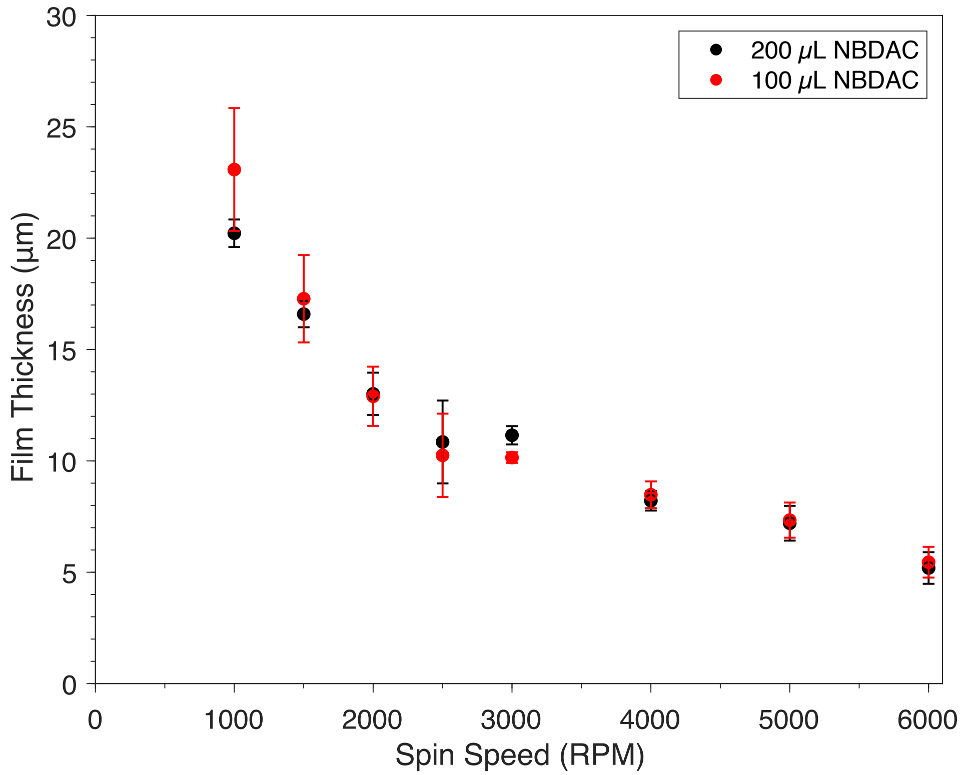

Figure S.5 Profilometric measurements of films made on Au using 100 and 200 μL of NBDAC. Points represent an average of n > 3 and error bars represent the standard deviation between samples.

SI.6 Calculation of Active Catalyst Concentration from GPC/SEC

To determine the amount of G3 that participates in the scROMP process, we assumed that each polymer chain contains only one catalyst molecule and that chain transfer is negligible[1]. The number of moles of catalyst used in the polymerization was then determined by

$$Mol_{G3}\ used = \frac{g_{polymer}}{M_{W_{polymer}}} \qquad (S.1)$$

where $g_{polymer}$ is the mass of the polymer synthesized and $M_{W,polymer}$ is the polymer molecular weight. These calculations were carried out for two systems. The first system was generated by dispensing 100 μL of G3 onto a static Au-coated wafer at various concentrations of G3 in DCM (Table S.1). Following solvent evaporation, a 1M solution of NB was dispensed onto the surface at a polymerization spin speed of 3000 RPM to yield pNB. The second system was generated by varying the spin speed during polymerization of NBDAC (Table S.2). First, a 5 mM G3 solution in DCM was dispensed onto a Au-coated wafer by spinning at 1000 RPM for 30 s. Then, neat liquid NBDAC monomer was dispensed on the surface at 1000, 2000, and 4000 RPM and allowed to polymerize for 60 s. After 60 s had elapsed, the surface was flooded with ethyl vinyl ether to terminate the polymerization reaction. The generated pNBDAC film was subsequently immersed into a 90/10 v/v% solution of THF/EtOH. This converted the acyl chloride side chains present on pNBDAC into ethyl ester side chains and yielded a THF soluble film that was then used for GPC/SEC analysis.

Table S.1. Molecular weight, polydispersity, and calculation of mols of G3 utilized during scROMP of NB with different amounts of G3 on the surface. All films were made using a 1M solution of NB in DCM spun on at 3000 RPM.

G3 Amount (nmol)	Polymer M_W (Da)	pNB PDI (Đ)	Polymer Mass (g)	G3 Used (nmol)
500	171000	1.42	0.0020	11.65
100	241400	1.19	0.0014	5.80
50	235800	1.17	0.0005	2.12

Table S.2 Calculation of mols of G3 utilized in the polymerization of NBDAC at various spin speeds.

Spin Speed (RPM)	Polymer M_w (Da)	Polymer Mass (g)	G3 Used (nmol)
1000	404600	0.0064	15.19
2000	480600	0.0040	7.93
4000	635900	0.0016	2.40

The results in Table S.1 indicate that the scROMP approach is capable of synthesizing polymer films with high M_W and low PDI, which is attributed to the fast initiation and propagation rates of G3. The polymer synthesized in the presence of 500 nmol of G3 is an extreme case where all catalyst dispensed on the surface while spinning would be retained. This perfect retention of catalyst is an unrealistic scenario for the scROMP approach and shows lower M_W and higher PDI due to the participation of excess catalyst in the polymerization. The polymers synthesized using 100 and 50 nmol G3 are more representative of the scROMP process. These polymers show higher

molecular weights and lower PDI, indicating that the scROMP approach can be used to produce well-defined polymers in ambient conditions with minimal solvent usage.

The results shown in Table S.2 demonstrate the impact that polymerization spin speed has on the resulting polymer M_W and amount of catalyst participating in the polymerization. As spin speed is increased, the amount of catalyst initiating and participating in propagation is decreased. We attribute the reduction in active G3 to higher shear stresses at the surface at increasing spin speeds. Polymer M_W increases with increasing spin speed, which we attribute to reduced competition between initiated catalysts to consume monomer for polymer chain propagation.

SI.7 ^{1}H NMR Spectra of p(NB-co-NBF4)

The ^{1}H NMR for pNB shown in Figure S.6 is consistent with literature spectra for pNB[2] and features a *trans* to *cis* ratio of 1:1.32. Referencing the notation used in Figure S.6., ($CDCl_3$): δ *cis*: (a) 5.21 (2H); (b) 2.78 (2H); (c, e) 1.83 (3H); (d) 1.35 (2H); (f) 1.03 (1H); *trans*: (g) 5.34 (2H); (h) 2.43 (2H); (i, k) 1.83 (3H); (j) 1.35 (2H); (l) 1.03 (1H). Moisture from the $CDCl_3$ appears at 1.55 ppm, and pentane absorbed in the film from dissolving the monomers appears at 1.30, 1.26, and 0.88 ppm. The ^{1}H NMR spectrum for pNBF4 is considerably less straightforward due to the varied placement of the perfluoro chain along the hydrocarbon backbone. For example, six different chemical shifts for just the olefinic protons could be observed from the conformations presented in Figure S.7. However, the peaks in Figure S.8. at 3.25 and 2.05 ppm only appear in the pNBF4 spectra and have been used to determine the relative composition of the monomer units within the film. We conclude that the 3.25 ppm peak represents the proton labeled x in the *cis* conformation of Fig. S.8., and the 2.05 ppm peak represents the c* and e* protons in the *cis* conformation and the i* and k* protons in the *trans* conformation. The integrations are referenced relative to the

olefinic area between 5.5 and 5.0 ppm, with the ratio between the 3.25 ppm peak and the 5.5-5.0 ppm area in the NBF4 representing a 100% pNBF4 composition. Values for composition deviated by <5% when using either the 3.25 or the 2.05 ppm peaks.

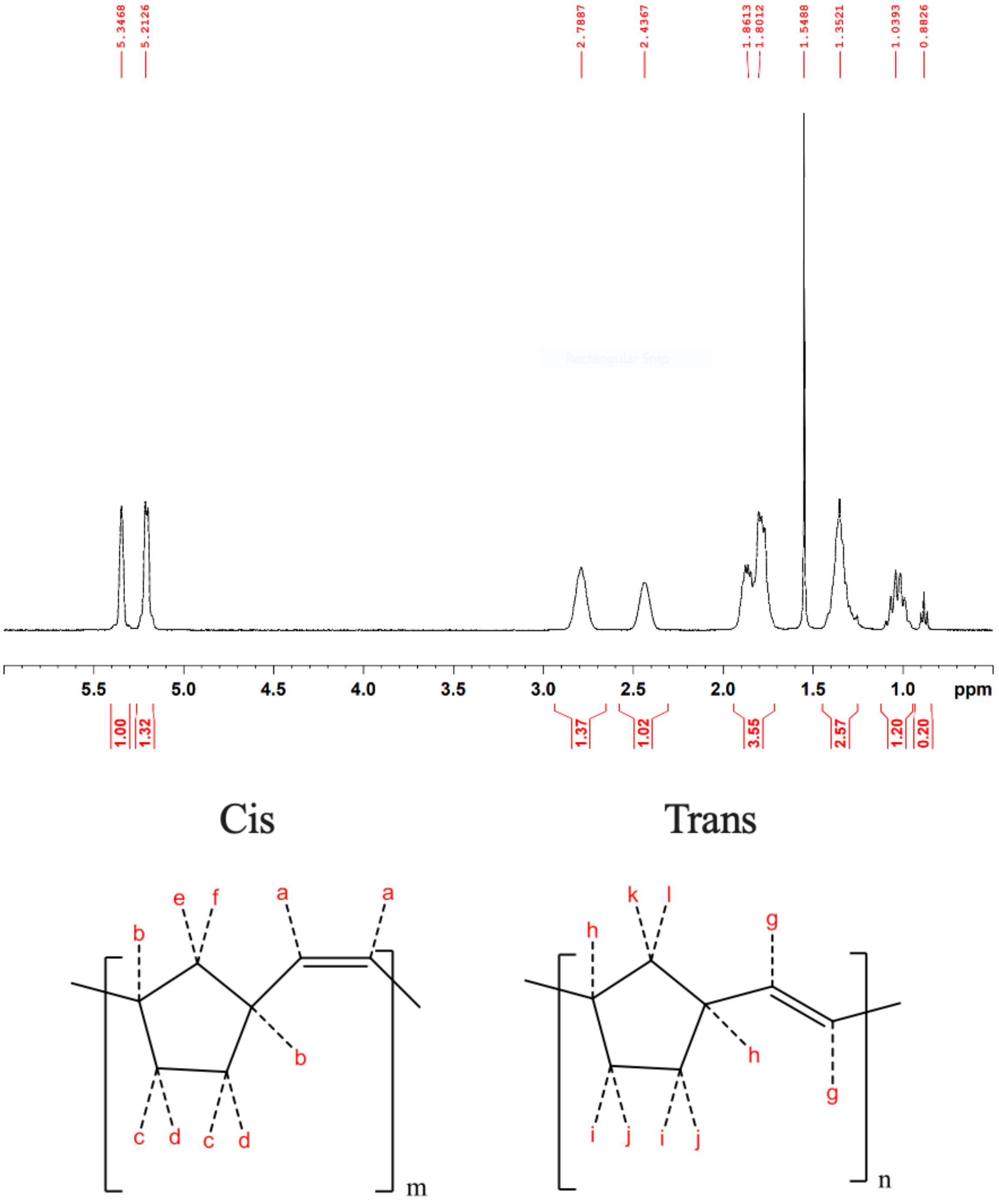

Figure S.6. ^{1}H NMR spectrum of pNB synthesized using scROMP.

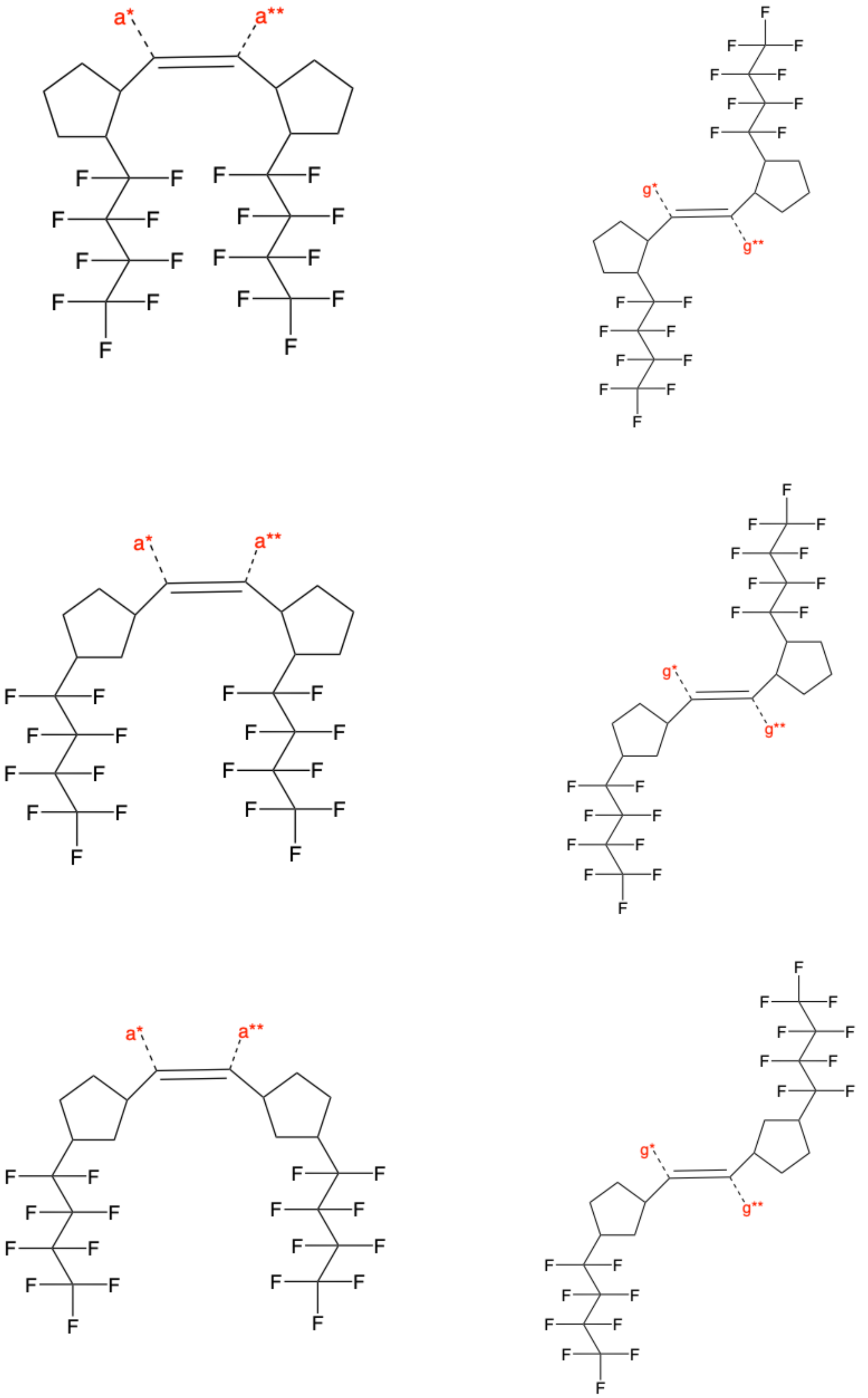

Figure S.7. Different conformations and placements of the fluorocarbon chain in pNBF4 affect the olefinic protons' chemical shifts.

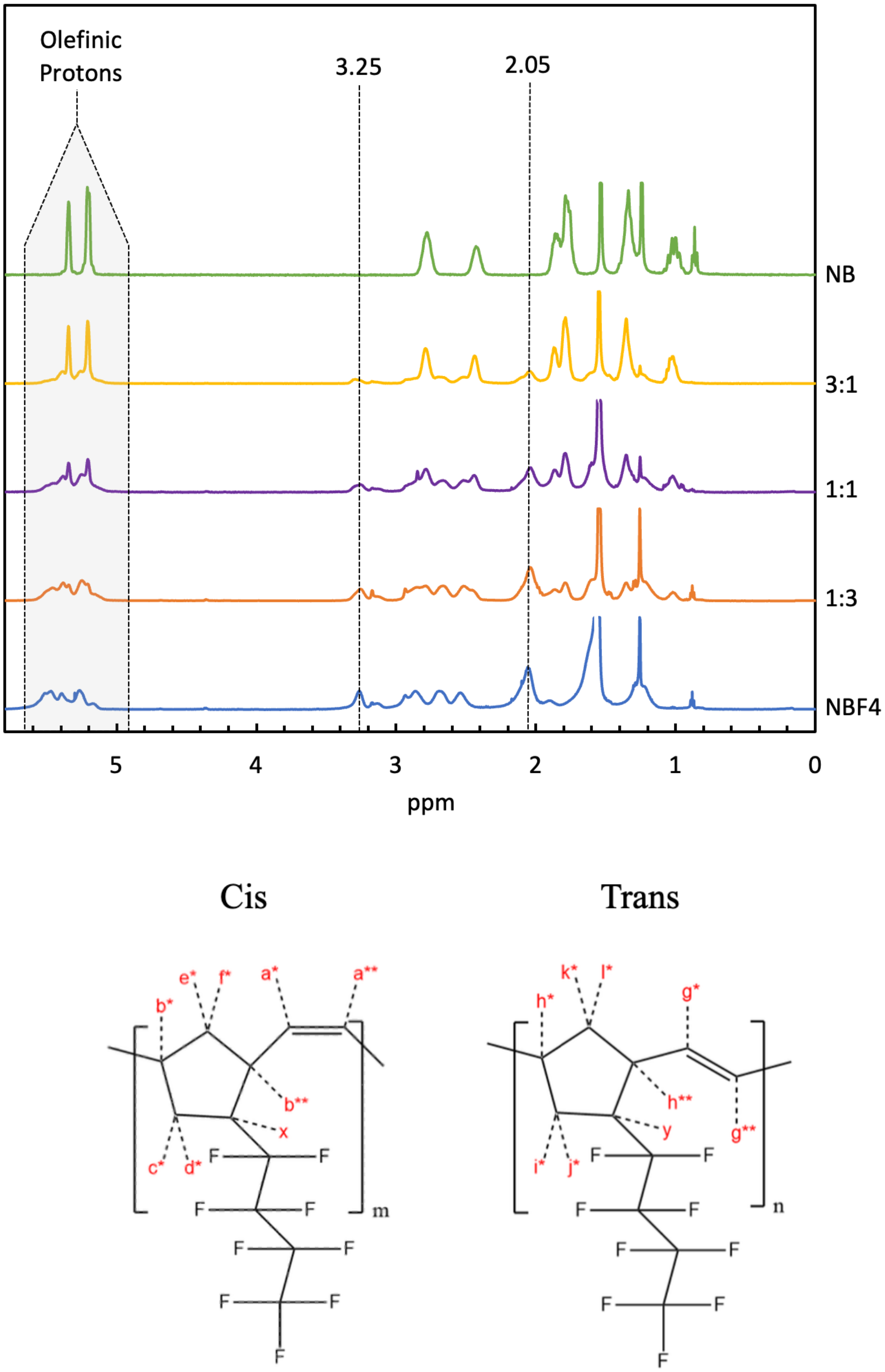

Figure S.8. ^{1}H NMR spectrum of p(NB-co-NBF4) films synthesized using scROMP. Ratios are referenced as monomer molar ratios of NB:NBF4.

Heteronuclear single quantum coherence (HSQC) spectroscopy experiments show the presence of green CH groups between 5.5 and 5.0 ppm as would be expected with olefinic protons. A series of green CH groups also appears between 3.5 and 2.5 ppm for the b*, b**, h*, h**, x, and y protons. The c*, d*, e*, f*, i*, j*, k*, and l* protons that are further away from the both the fluorocarbon and olefin regions appear as red CH_2 in the less deshielded region.

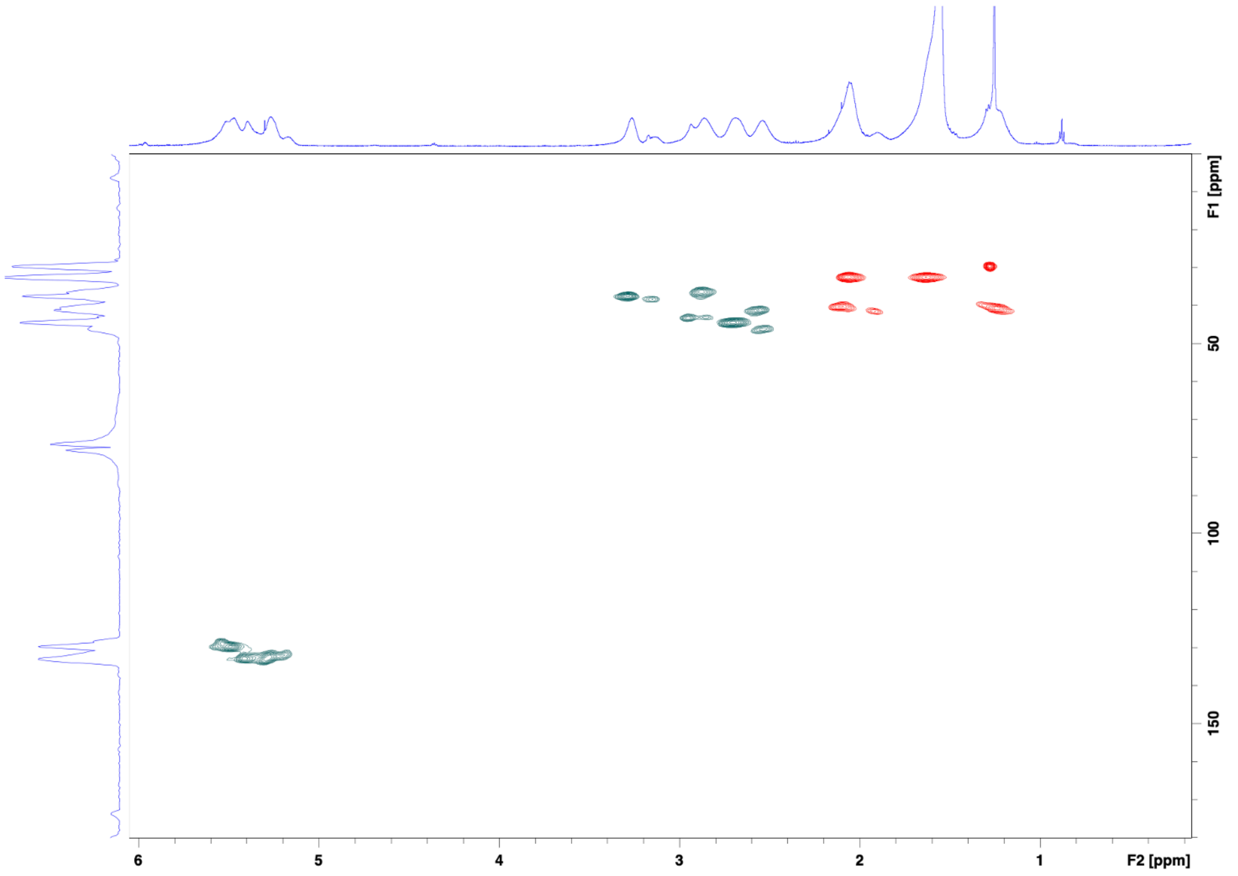

Figure S.9. HSQC for the pNBF4 film synthesized using scROMP with axis F2 showing the ^{1}H NMR. CH/CH_3 signals are phased positive and in green, and CH_2 signals are phased negative and in red.

SI.8 GPC/SEC Data of p(NB-co-NBF4) Polymers

Polymer chain lengths and molecular weight distributions for p(NB-co-NBF4) were determined using GPC. The degree of polymerization (DP) was determined by dividing the mass-average molecular weight (M_W) by the molar mass of the repeat unit, resulting in the average number of repeat units per chain. For copolymers, DP was determined through the same approach using the NB:NBF4 NMR ratios presented in Table 3 of the main text. The equation for DP calculation of copolymers is written as:

$$DP = \frac{M_W}{(mol\%\ NB)(M_{W_{NB}}) + (mol\%\ NBF4)(M_{W_{NBF4}})} \quad (S.2)$$

Both number- and weight-average molecular weights increased with fluorocarbon concentration, while polydispersity and degree of polymerization decreased. Increases in average molecular weights were expected since the NBF4 repeat unit is ~3x the molecular weight of a NB repeat unit. The degree of polymerization decreased with increasing NBF4 monomer concentration; however, NMR data indicate that the NBF4 monomer is more rapidly incorporated into the polymer film than the NB monomer is. The presence of the NBF4 must hinder the polymerization of NB enough to decrease the overall degree of polymerization in monomer systems with high NBF4 ratios. The fluorocarbon and hydrocarbon monomers are largely incompatible, so the NB may be repelled from the catalyst, decreasing the overall number of monomers polymerized. The polydispersity index is lower for systems with high amounts of NBF4 since the presence of the fluorocarbon chain may decrease the likelihood for chain terminations, such as backbiting by the catalyst,[3] by screening susceptible olefinic bonds.

Table S.3. Polydispersity and molecular weights of p(NB-co-NBF4) films using GPC. Degree of polymerization was calculated from M_w and ^1H NMR data. Films synthesized using ratios less than 1:1 were not soluble enough in THF to produce meaningful signals.

Monomer Ratio (NB:NBF4)	Polydispersity (PDI)	Number-Average Molecular Weight (M_n)	Weight-Average Molecular Weight (M_w)	Degree of Polymerization (DP)
1:1	1.17	2.73×10^5	3.20×10^5	1.37×10^3
3:1	1.24	2.49×10^5	3.10×10^5	1.94×10^3
NB	1.42	1.49×10^5	2.12×10^5	2.25×10^3

SI.9 Membrane Pervaporation Testing Setup

Membranes were tested in a pervaporation setup. A schematic of the equipment train is shown below in Scheme S.1. Briefly, a solution of 90/10 v/v% EtOH/H_2O was added to a feed vessel. The feed vessel was placed onto a heated stir plate and heated to 55 ºC. A rotary pump was used to circulate the heated feed into the membrane cell and back into the feed tank. After the feed reached the desired temperature, a vacuum pump connected to a cold trap on the permeate side was powered on. CAUTION: Vacuum should be powered on before cooling the cold trap with liquid N_2 to prevent hazardous liquid oxygen formation. After the permeate side was under vacuum, the cold trap was placed into a dewar filled with liquid nitrogen.

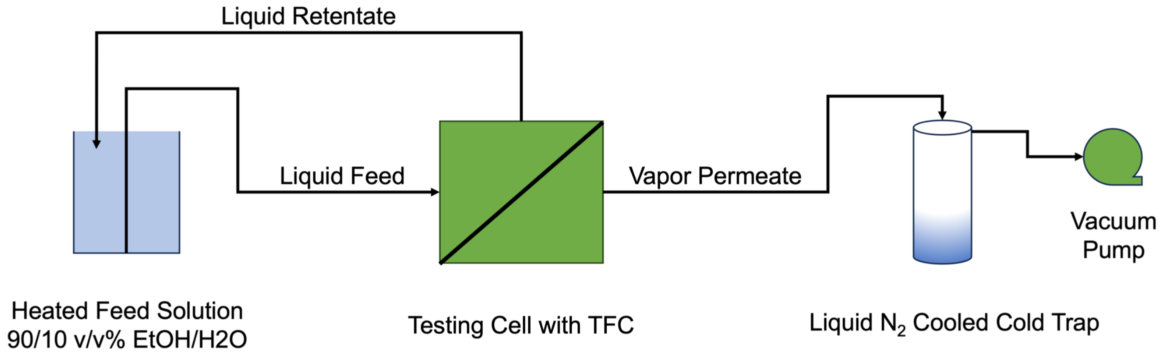

Scheme S.1. Schematic of membrane pervaporation setup used for ethanol dehydration.

References

(1) Bielawski, C. W.; Grubbs, R. H. Living Ring-Opening Metathesis Polymerization. *Progress in Polymer Science* **2007**, *32* (1), 1–29. https://doi.org/10.1016/j.progpolymsci.2006.08.006.

(2) Autenrieth, B.; Schrock, R. R. Stereospecific Ring-Opening Metathesis Polymerization (ROMP) of Norbornene and Tetracyclododecene by Mo and W Initiators. *Macromolecules* **2015**, *48* (8), 2493–2503. https://doi.org/10.1021/acs.macromol.5b00161.

(3) Harada, Y.; Girolami, G. S.; Nuzzo, R. G. Catalytic Amplification of Patterning via Surface-Confined Ring-Opening Metathesis Polymerization on Mixed Primer Layers Formed by Contact Printing. *Langmuir* **2003**, *19* (12), 5104–5114. https://doi.org/10.1021/la020924v.
